# Comparison of the Bioactive Components in Two Seeds of *Ziziphus* Species by Different Analytical Approaches Combined with Chemometrics

**DOI:** 10.3389/fphar.2017.00609

**Published:** 2017-09-05

**Authors:** Sheng Guo, Jin-Ao Duan, Yiqun Li, Ruiqing Wang, Hui Yan, Dawei Qian, Yuping Tang, Shulan Su

**Affiliations:** Jiangsu Collaborative Innovation Center of Chinese Medicinal Resources Industrialization, State Key Laboratory Cultivation Base for TCM Quality and Efficacy, Nanjing University of Chinese Medicine Nanjing, China

**Keywords:** *Ziziphus jujuba* var. *spinosa*, *Ziziphus mauritiana*, saponins, flavonoids, fatty acids, chemometrics

## Abstract

The *Ziziphus* species are considered to be the medicine and food dual purposes plants. Among them, the seed of *Ziziphus jujuba* var. *spinosa* (ZS) has traditionally been used as an ethnomedicine in Asian countries for thousands years. Owing to the significant benefits for human health, the demand for ZS increased year by year, and the wild resources have become increasingly scarce, which resulted in a shortage of market supply for ZS and product adulteration by substituting ZS with the seeds of *Z. mauritiana* Lam. (ZM). However, whether the bioactivity of ZM is similar to ZS has not been fully confirmed till now. Thus, to provide potential information for evaluating the similarity of the health promoting activities between these two *Ziziphus* seeds, their chemical profiles, including triterpenoids, flavonoids, nucleosides, free amino acids and fatty acids were compared using high-performance liquid chromatography coupled with evaporative light scattering detection (HPLC-ELSD), ultra-high performance liquid chromatography coupled with triple-quadrupole mass spectrometry (UHPLC-TQ MS), and gas chromatography coupled with mass spectrometry (GC-MS) methods. Furthermore, a more holistic investigation was performed with multivariate principle component analysis and orthogonal projections to latent structures-discriminant analysis analyses to explore the relative variability between the seeds of two species. The results showed that a significant difference exists between ZS and ZM, and ZS was more rich in saponins, polyunsaturated fatty acids and some amino acids, whereas ZM was particularly rich in saturated fatty acids and flavonoids. The above results suggested the bioactivities of ZM for human health may not be similar to ZS owing to their difference in chemical profiles. These results would also be helpful for distinguishing the ZM from ZS with the chemical markers obtained from the study, and set a scientific foundation for establishing the quality control method of ZS.

## Introduction

*Ziziphus* species (Rhamnaceae family) are considered to be multipurpose plants and have been used as foods, folk medicines, environmental protection plants, etc., which are distributed mainly in warm and subtropical regions throughout the world (Guo et al., 2011b). As an important economic species, *Ziziphus jujuba* Mill. var. *spinosa* (Bunge) Hu ex H. F. Chou has generated much commercial value due to its beneficial effects for human health and significant pharmacological activities, and its fruit is widely consumed as part of the folk medicines in eastern Asia (Sun et al., 2011; Yang et al., 2013). Its seeds (ZS), called Suan Zao Ren in Chinese, have been used over two thousand years in China as crude drugs in TCMs for the treatment of insomnia and anxiety with a long term effect on sleep architecture and low toxicity. Modern pharmacological studies have demonstrated that ZS possesses multiple activities such as hypnotic effects (Cao et al., 2010), sedative effects (Fang et al., 2010), antidepressant-like effects (Liu et al., 2012), learning improvement and memory enhancement effects (Zhang et al., 2014), and so on (Lee et al., 2008; Chen et al., 2009; Xie et al., 2012). Phytochemical studies on ZS resulted in the isolation and identification of flavonoids (Cheng et al., 2000; Wu et al., 2015), triterpene acids and their saponins (Ma et al., 2011; Wang et al., 2013; Wu et al., 2013), alkaloids (Kang et al., 2016), indole derivatives (Li et al., 2014), and fatty acids (Zhao et al., 2006). Among them, saponins, flavonoid *C*-glycosides and fatty oil with abundance of unsaturated fatty acids in ZS are considered as important for sedative and hypnotic effects (Jiang et al., 2007; Xie et al., 2012), through a variety of mechanisms and targets, especially 5-HT metabolism, GABAergic receptors and hippocampal neurons (Wang et al., 2010; You et al., 2010; Wang et al., 2012). In addition, amino acids and nucleosides contained in *Ziziphus* plants are valuable nutrients and reportedly involved in the regulation and modulation of various physiological processes in the body, which all contribute to human health (Guo et al., 2013, 2015).

In recent years, owing to its significant beneficial values for human health, the demand for ZS has increased year by year, and the wild resources become increasingly scarce. The above fact resulted in a shortage of market supply for ZS and product adulteration by substituting ZS with the seeds of *Z. mauritiana* Lam. and *Hovenia acerba* Lindl. (Sun et al., 2014). Among these two adulterants, the former, obtained from the same genus with the genuine ZS, was more prevalent in the market. This phenomenon could be owing to the fact that this species is widely cultivated in China for its edible fruit and it is plausible that its seed (normally discarded) has been used here as a cheap look-alike substitute for the official drug (Zhang et al., 2016; Van der Valk et al., 2017). It was reported that the seeds of *Z. mauritiana* (ZM) is also used as folk medicine in Yunnan province and surrounding areas in China with similar health promoting effect to ZS. However, whether its bioactivities are similar to ZS has not been fully investigated till now. In recent years, many qualitative studies were performed on ZS samples, and mainly focused one or two types of chemical structures (Zhang et al., 2008; Kim et al., 2014; Zhao et al., 2014). However, there has not been a systematic comparison of chemical profiles between the seeds of the two *Ziziphus* species.

In order to provide information on the similarity of the health promoting activities between these two *Ziziphus* seeds through the chemical profile comparison, triterpenoids, flavonoid *C*-glycosides, nucleosides, free amino acids and fatty acids, a total of 59 compounds were analyzed in this paper. Furthermore, a multivariate statistical method was used to discover and evaluate the chemical differences between them.

## Materials and Methods

### Chemicals

Reference compounds of spinosin, jujuboside A and betulinic acid were purchased from the National Institute for the Control of Pharmaceutical and Biological Products (Beijing, China). Chemical standards of 6″′-feruloylspinosin and jujuboside B were obtained from Aladdin Chemical Co. (Nanjing, China). The chemical standards of amino acids (phenylalanine, leucine, isoleucine, tryptophan, cysteine, methionine, taurine, proline, valine, tyrosine, γ-aminobutyric acid, hydroxyproline, alanine, threonine, lysine, glutamine, serine, asparagine, citrulline, glutamic acid, aspartic acid, histidine, arginine, ornithine), nucleosides and nucleobases (thymine, thymidine, 2′-deoxyuridine, 2′-deoxyadenosine, adenine, uridine, hypoxanthine, adenosine, 2′-deoxyinosine, cytosine, inosine, guanine, cytidine, guanosine, 2′-deoxyadenosine-5′-monophosphate, adenosine-5′-monophosphate, inosine-5′-monophosphate, guanosine-5′-monophosphate, cytidine-5′-monophosphate) were from Sigma Chemical Co. (St. Louis, MO, United States). The purity of each compound was above 98%. The chemical structures of these reference compounds are shown in Supplementary Figure S1. The standard mixture of 37 fatty acid methyl esters (FAME) used to establish calibration curves was purchased from ANPEL Scientific Instrument Co. (Shanghai, China).

Acetonitrile (HPLC-grade) was purchased from Merck (Darmstadt, Germany), and deionized water (H_2_O) was purified by a Milli-Q water purification system (Millipore, Billerica, MA, United States). Other reagent solutions, such as ammonium acetate and acetic acid, were of analytical grade (SinoPharm Chemical Reagent Co., Ltd., Shanghai, China).

Twelve batches of *Ziziphus jujuba* var. *spinosa* seeds (ZS01∼12) were collected from different habitats in September 2014, and seven batches of *Z. mauritiana* seeds (ZM01∼07) were collected from different markets which were all imported from Southeast Asia. The information on these samples is summarized in **Table 1**. Their botanical origins were identified as *Z. jujuba* Mill. var. *spinosa* (Bunge) Hu ex H. F. Chou and *Z. mauritiana* Lam., respectively, by the corresponding author. Voucher specimens were deposited at the Herbarium at the Nanjing University of Chinese Medicine, China.

**Table 1:** Contents of flavonoids and triterpenoids (mg/100 g) in the seeds of *Ziziphus jujuba* var. *spinosa* and *Z. mauritiana* (mean ± SD).

Sample number	Collecting region	Spinosin	6″′-feruloyl-spinosin	Jujuboside A	Jujuboside B	Betulinic acid
**Seeds of *Ziziphus jujuba* var. *spinosa***					
ZS01	Pingyi, Shandong	84.02 ± 1.16^f^	22.22 ± 0.52^c^	79.66 ± 2.49^d^	29.04 ± 1.02^h^	38.56 ± 1.11^b^
ZS02	Fuxin, Liaoning	77.88 ± 2.24^e^	42.20 ± 1.06^f^	70.84 ± 1.88^c^	19.63 ± 0.51^ef^	63.76 ± 2.05^d^
ZS03	Chaoyang, Liaoning	61.20 ± 2.65^c^	21.55 ± 0.43^c^	37.12 ± 1.04^a^	14.64 ± 0.45^c^	31.54 ± 1.12^a^
ZS04	Zanhuang, Hebei	21.98 ± 1.04^a^	15.32 ± 0.50^b^	74.78 ± 1.49^cd^	11.89 ± 0.35^b^	36.24 ± 1.53^ab^
ZS05	Turpan, Xinjiang	89.36 ± 2.21^g^	26.07 ± 0.69^d^	76.55 ± 3.08^cd^	10.05 ± 0.32^a^	37.37 ± 1.09^ab^
ZS06	Neiqiu, Hebei	31.29 ± 0.64^b^	11.66 ± 0.35^a^	89.03 ± 2.17^e^	19.20 ± 0.59^ef^	51.91 ± 2.01^c^
ZS07	Suide, Shaanxi	25.94 ± 0.55^a^	26.52 ± 0.53^d^	55.05 ± 1.41^b^	13.71 ± 0.39^c^	72.41 ± 2.01^e^
ZS08	Jiaxian, Shaanxi	90.51 ± 1.04^g^	42.56 ± 1.71^f^	78.47 ± 2.68^d^	20.59 ± 0.73^f^	50.27 ± 1.80^c^
ZS09	Yanan, Shaanxi	69.84 ± 1.63^d^	39.55 ± 1.40^f^	59.15 ± 1.89^b^	18.17 ± 0.49^e^	55.30 ± 2.28^c^
ZS10	Xingtai, Hebei	79.25 ± 2.67^ef^	35.78 ± 1.43^e^	90.34 ± 2.81^e^	13.69 ± 0.50^c^	90.51 ± 3.37^f^
ZS11	Zibo, Shandong	113.64 ± 1.83^h^	45.75 ± 1.22^g^	114.08 ± 3.41^f^	16.43 ± 0.40^d^	92.57 ± 2.86^f^
ZS12	Yinchuan, Ningxia	132.38 ± 2.23^i^	40.48 ± 1.53^f^	125.41 ± 4.05^g^	23.80 ± 0.76^g^	89.41 ± 3.17^f^
mean		73.11 ± 33.90	30.81 ± 11.65	79.21 ± 24.17	17.57 ± 5.35	59.15 ± 22.45
**Seeds of *Ziziphus mauritiana***					
ZM01	Anguo, Hebei	107.48 ± 2.75^d^	34.47 ± 1.21^d^	nd	nd	31.47 ± 0.94^a^
ZM02	Bozhou, Anhui	128.22 ± 3.55^e^	42.71 ± 1.02^e^	nd	nd	49.52 ± 1.57^c^
ZM03	Kunming, Yunnan	78.84 ± 1.49^b^	29.26 ± 1.26^c^	nd	nd	27.35 ± 0.73^a^
ZM04	Yuanmou, Yunnan	94.04 ± 3.34^c^	18.59 ± 0.52^a^	nd	nd	31.09 ± 1.28^a^
ZM05	Xian, Shaanxi	67.64 ± 2.71^a^	20.96 ± 0.39^ab^	nd	nd	48.50 ± 1.72^c^
ZM06	Nanjing, Jiangsu	124.88 ± 3.26^e^	48.91 ± 1.25^f^	nd	nd	92.60 ± 3.21^d^
ZM07	Haikou, Hainan	62.97 ± 1.96^a^	23.23 ± 0.72^b^	nd	nd	40.01 ± 1.19^b^
Mean		94.86 ± 26.42	31.16 ± 11.46	nd	nd	45.79 ± 22.39
*P*^#^		^∗^	ns	^∗∗^	^∗∗^	^∗^

*Means within a column with different superscripts differ significantly (*P* < 0.05). nd, not detected. ^#^*P*-value for Seeds of *Ziziphus jujuba* var. *spinosa* × Seeds of *Ziziphus mauritiana* (ns, not significant); ^∗^*P* < 0.05, ^∗∗^*P* < 0.01.*

### Analysis of Flavonoids and Triterpenoids

Flavonoids and triterpenoids were analyzed on a Waters 2695 Alliance HPLC system (Waters Corp., Milford, MA, United States), which includes a quaternary pump solvent management system, an online degasser, and an autosampler. The samples were pulverized to homogeneous powders (40 mesh) and about 1.0 g of the above powder was weighed accurately into a 50 mL conical flask with a stopper, and extracted with 20 mL of 70% methanol aqueous solution in a cooled ultrasonic bath (40 kHz) for 30 min. The solution was centrifuged at 8000 × *g* for 10 min. The supernatant was separated and 20 μL of sample was injected into an Alltima C18 column (250 mm × 4.6 mm, 5 μm). The mobile phase was composed of A (Acetonitrile) and B (0.1% formic acid in H_2_O, *v*/*v*) with a gradient elution: 0–26 min, 16–27%A; 26–27 min, 27%A–36%A; 27–41 min, 36%A–39%A; 41–45 min, 39–90% A; 45–55 min, 90–100% A; 55–60 min, 100% A. The flow rate of the mobile phase was maintained at 1.0 mL/min. A Waters 2424 ELSD was used as a detector and the raw data was acquired and processed with Empower software. The drift tube temperature of the ELSD was set at 80°C and the nitrogen flow rate was 2.7 L/min. Quantification was performed according to the linear calibration plots of the logarithm of peak areas vs. the logarithm of concentration. The validation of the method was performed as described in Supplementary Material. The concentrations were expressed in milligrams per 100 g of dry weight (DW) from three replications.

### Analysis of Amino Acids, Nucleosides, and Nucleobases

Amino acids, nucleosides and nucleobases were analyzed on a UPLC-MS/MS system (ACQUITY UPLC, Xevo TQ tandem quadrupole mass spectrometer; Waters Corporation; Milford, MA, United States) with a modified procedure from the reference (Ge et al., 2016). A total of 1.0 g of sample powder (40 mesh) was extracted with 20 mL of water in a cooled ultrasonic bath (40 kHz) for 60 min, followed by centrifugation (15000 × *g*, 10 min). The supernatant was filtered thought a 0.22 μm filter, then the 1 μL of sample was injected into an ACQUITY UPLC BEH Amide column (2.1 mm × 100 mm, 1.7 μm) operated at 35°C. The mobile phase was composed of A (5 mmol/L ammonium acetate, 5 mmol/L ammonium formate and 0.2% formic acid in deionized water) and B (1 mmol/L ammonium acetate, 1 mmol/L ammonium formate and 0.2% formic acid in acetonitrile) with a gradient elution (0–3 min, 10% A; 3–9 min, 10–18% A; 9–15 min: 18–20% A; 15–16 min, 20–46% A; 16–20 min, 46% A). The flow rate of the mobile phase was 0.4 mL/min. The column eluent was directed to the mass spectrometer with an ESI source. The parameters were set as the reference with a slight modification (Ge et al., 2016), and the selected values of cone voltage and collision energy for each target compound are presented in Supplementary Table S1. Concentrations of the target compounds were calculated from the peak areas of the sample and the corresponding standards. The validation of the method was performed as described in Supplementary Material. The concentrations were expressed in micrograms per 100 g of DW from three replications.

### Analysis of Fatty Acids

The fatty acid profile was determined after a transesterification procedure according to EU standard methods (Annexes II and IX of European Community Regulation EEC/2568/91). A total of 1.0 g of sample powder (40 mesh) was extracted with 20 mL of *n*-heptane in a cooled ultrasonic bath (40 kHz) for 30 min, followed by centrifugation (3000 × *g*, 10 min). The supernatant was used for the cold alkaline transesterification with methanolic potassium hydroxide (KOH) solution, and the methyl esters (FAME) were extracted with *n*-heptane. The above solution was diluted to 1:10 or 1:100 with *n*-heptane, as necessary to give the sample working solutions. An aliquot (1 μL) of the FAME working solutions was used for the determination with a gas chromatograph (PerkinElmer Clarus 680, United States). The separation was performed on a HP-5 MS capillary column (PerkinElmer, 30 m × 250 μm, 0.25 μm film thickness). The oven temperature was programmed at 50°C during the first 4 min, increasing to 140°C at 10°C/min and holding for 4 min, then rising to 180°C at 2°C/min, finally increasing to 225°C at 1°C/min and holding for 3 min. The temperatures of the injector and detector were 200 and 250°C, respectively. The split ratio was 1:20 and helium was used as carrier gas. The column eluent was directed into an AxION-iQT mass spectrophotometer (PerkinElmer, United States) with an EI source. The source temperature was set at 250°C, collision energy at 70 eV, and the analyte detection were performed by using select ion monitoring (SIM) as presented in Supplementary Table S2. Methyl salicylate was selected as the internal standard which was used for the normalization of the chromatographic peak area of each sample. Identification and quantification was conducted by comparing the retention time, peak area and MS spectra with 37 standards of FAME. The validation of the method was performed as described in Supplementary Material. The results were expressed as mg/g of DW.

### Statistical Analysis

The results were reported as mean ± standard deviation (SD) of triplicate determinations. Data were statistically evaluated using one-way ANOVA analysis and the Least Significant Difference (LSD) test was used to compare mean values between samples from the different regions (level of significance: *p* < 0.05). Moreover, the overall differences between the samples from *Z. jujuba* var. *spinosa* and *Z. mauritiana* were also studied by ANOVA. Data detected in the samples were subjected to principle component analysis (PCA) and a general overview of the variance of metabolites was obtained. All the above statistical calculations were carried out with the aid of SPSS 18.0 software (SPSS, Chicago, IL, United States). Orthogonal projections to latent structures-discriminant analysis (OPLS-DA) was performed with the program SIMCA-P Version 14.1 (Umetrics, Umea, Sweden). The biomarkers for distinguishing the two species were subsequently identified by analyzing the S-plot, which was declared with covariance (*p*) and correlation (*pcor*). All variables were mean centered and scaled to Pareto variance.

## Results and Discussion

### Flavonoids and Triterpenoids

**FIGURE 1:**
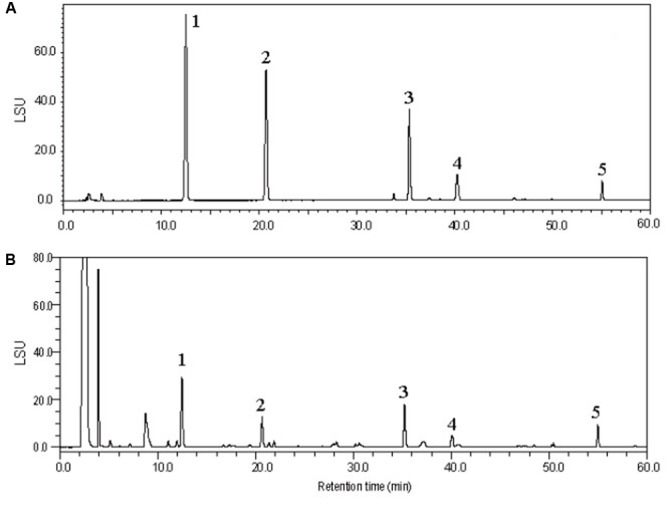
High-performance liquid chromatography coupled with evaporative light scattering detection (HPLC-ELSD) chromatography of mixed standards **(A)** and sample **(B)** for flavonoids and triterpenoids analyzed in the present study. Spinosin (1), 6″′-feruloylspinosin (2), jujuboside A (3), jujuboside B (4), and betulinic acid (5).

Two flavonoids (spinosin and 6″′-feruloylspinosin) and three triterpenoids (jujuboside A, jujuboside B and betulinic acid) were analyzed with an high-performance liquid chromatography coupled with evaporative light scattering detection (HPLC-ELSD) method which was validated by determination of the linearity, limit of detection (LOD) and quantitation (LOQ), intra-day and inter-day precisions, stability and accuracy. The results are summarized in Supplementary Tables S3, S4. The typical chromatograms of the reference compounds and the sample are presented in **Figure 1**. **Table 1** showed the analysis results of the 19 batches of samples. For the flavonoids, it was shown that the average content of spinosin in ZM (0.95 mg/g) was higher than that in ZS (0.73 mg/g), while no significance was found for 6″′-feruloylspinosin. As for the triterpenoids, the average contents of jujuboside A and jujuboside B in ZS were 0.79 and 0.18 mg/g, respectively, while their levels in ZM were too low to be detected. Betulinic acid showed the similar trend with higher content in ZS than ZM. For the samples collected in different habitats, the differences among the contents of analytes were also found, consistent with previously published reference report (Zhao et al., 2014). In general, the samples of ZS11 and ZS12 collected from Yinchuan (Ningxia) and Zibo (Sandong), respectively, exhibited higher levels of flavonoids and triterpenoids than other ZS samples. Differences according to the collection sites were also found in ZM samples, in which the highest content of spinosin was 1.25 mg/g (ZM06), while the lowest was only about 0.63 mg/g (ZM07), nearly two times of the difference being found.

To date, triterpenoid saponins and flavonoids in the ZS samples are popularly acknowledged to be the bioactive constituents, especially for the saponins which have more effective sedative and hypnotic functions (Jiang et al., 2007; Cao et al., 2010). Thus, due to being poor in jujuboside A and B as revealed in present study, ZM samples could be considered as inferior compared to the ZS samples with regard to the important roles that saponins play in the pharmacological actions.

### Amino Acids, Nucleosides, and Nucleobases

**FIGURE 2:**
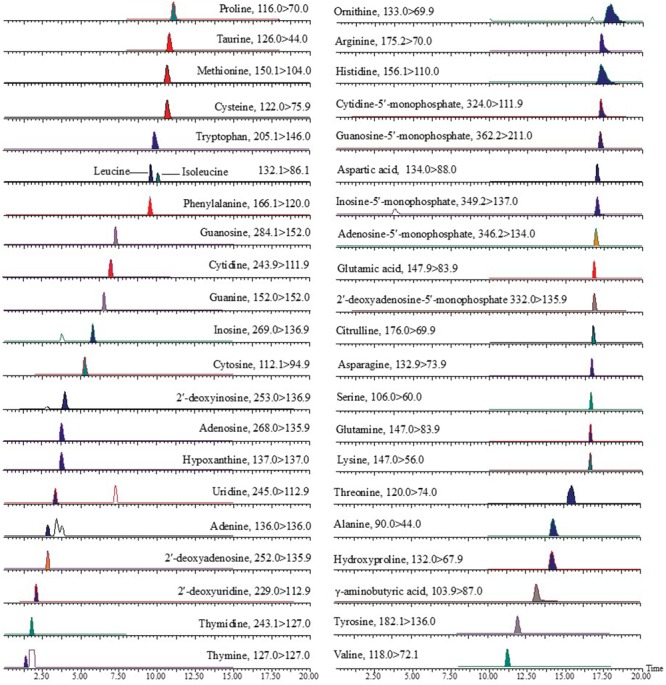
Ultra-high performance liquid chromatography coupled with triple-quadrupole mass spectrometry (UHPLC-TQ MS) chromatography (MRM/SIM) of mixed standards for amino acids, nucleosides, and nucleobases analyzed in the present study.

**FIGURE 3:**
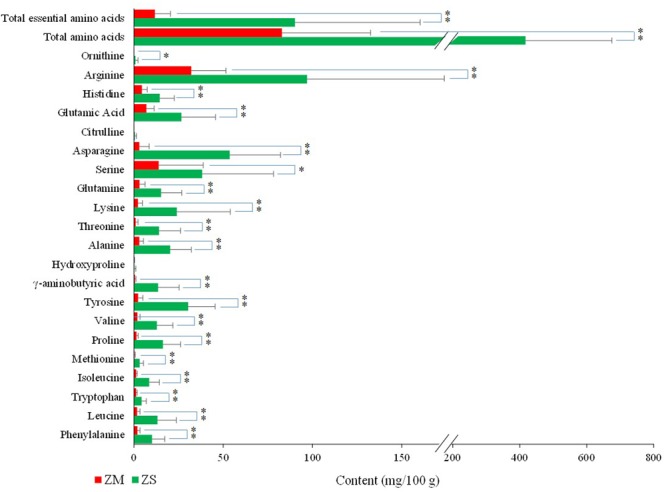
Contents of free amino acids (mg/100 g) in the seeds of *Ziziphus jujuba* var. *spinosa* (ZS) and *Z. mauritiana* (ZM). ^∗^*P* < 0.05, ^∗∗^*P* < 0.01.

Amino acids, nucleosides, and nucleobases were analyzed on a UPLC-MS/MS system with a modified procedure from the reference (Ge et al., 2016), and the typical chromatograms of the standards are presented in **Figure 2**. The results of the method were validated by determination of the linearity, LOD, LOQ, intra-day and inter-day precisions, stability, and accuracy. All the results are summarized in Supplementary Table S5, which showed that this UPLC-MS/MS method could be suitable for determination of the amino acids, nucleosides and nucleobases in the samples. A total of 21 amino acids, including 4 non-protein amino acids (γ-aminobutyric acid, hydroxyproline, citrulline, ornithine) were detected in ZS samples, and total 19 amino acids including 2 non-protein amino acids (γ-aminobutyric acid, hydroxyproline) were detected in ZM samples (Supplementary Table S6 and **Figure 3**). The total contents of these amino acids ranged from 1.40 to 9.23 mg/g for ZS samples and from 0.17 to 1.38 mg/g for ZM samples. The average content of total amino acids in ZS samples was found as 4.17 mg/g, which was significantly higher than that of ZM samples (0.83 mg/g). Similarly, a difference was also found for the total contents of essential amino acids which average content in ZS samples was 0.90 mg/g, nearly eight times of that in ZM samples (0.12 mg/g). For most of ZS samples, arginine, asparagine, serine and tyrosine were found with relatively high contents, and their average contents were 0.97, 0.54, 0.38, and 0.30 mg/g, respectively, which accounted for approximately 50% of the total amino acids. The main amino acids for most of ZM samples were found to be arginine (0.32 mg/g) and serine (0.14 mg/g). It is noteworthy that γ-aminobutyric acid, a non-protein amino acid with biological activities and potential health benefits, is present with high concentration in ZS samples, and its average level reached 0.14 mg/g, more than 10 times higher than that in ZM samples. In addition, the average content of proline in ZS (0.162 mg/g) is far greater than that in ZM (0.012 mg/g). It was reported that proline is closely related with the plant stress tolerance and the higher the content, the stronger resistance of the plants was, especially with respect to drought resistance (Krasensky and Jonak, 2012). *Z. jujuba* var. *spinosa* is mainly distributed in the northern area of China with an arid climate, which makes the plant develop strong drought tolerance. Whereas, *Z. mauritiana* is mainly distributed in Southeast Asia with the hot and humid climate, which could make the plant poor drought resistance and low proline content. It was also found that the difference in the levels of these compounds was existed among the samples obtained from different production areas for both ZS and ZM samples.

**FIGURE 4:**
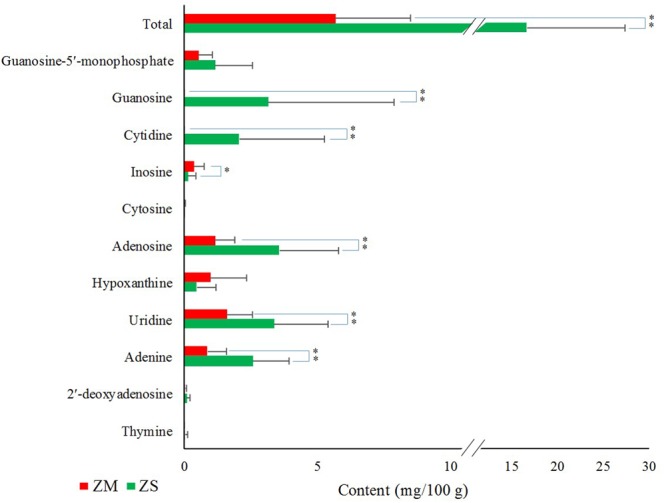
Contents of nucleosides and nucleobases (mg/100 g) in the seeds of *Z. jujuba* var. *spinosa* (ZS) and *Z. mauritiana* (ZM). ^∗^*P* < 0.05, ^∗∗^*P* < 0.01.

As for the nucleosides and nucleobases analyzed in the assay, a total of 11 and 8 analytes were detected in ZS and ZM samples, respectively (Supplementary Table S7 and **Figure 4**). The total contents of these nucleosides and nucleobases ranged from 37.8 to 345.3 μg/g for ZS samples and from 2.17 to 11.19 μg/g for ZM samples. The average content of total nucleosides and nucleobases in ZS samples (167.2 μg/g) was significantly higher than that of ZM samples (56.8 μg/g), and the difference was also found for the individual compounds. Adenosine, uridine and adenine were found with relatively high content in most of ZS samples, and their average contents were 35.8, 33.9, and 25.8 μg/g, respectively, which accounted for nearly 60% of the total content of nucleosides and nucleobases. The main nucleosides and nucleobases for most of ZM samples were found to be uridine (16.2 μg/g) and adenosine (11.6 μg/g). It was also found that the contents of guanosine and cytidine greatly varied among the samples, and their contents were found as high as 131.3 and 106.5 μg/g, respectively, while in some samples, especially ZM, they could not be detected.

### Fatty Acids

**FIGURE 5:**
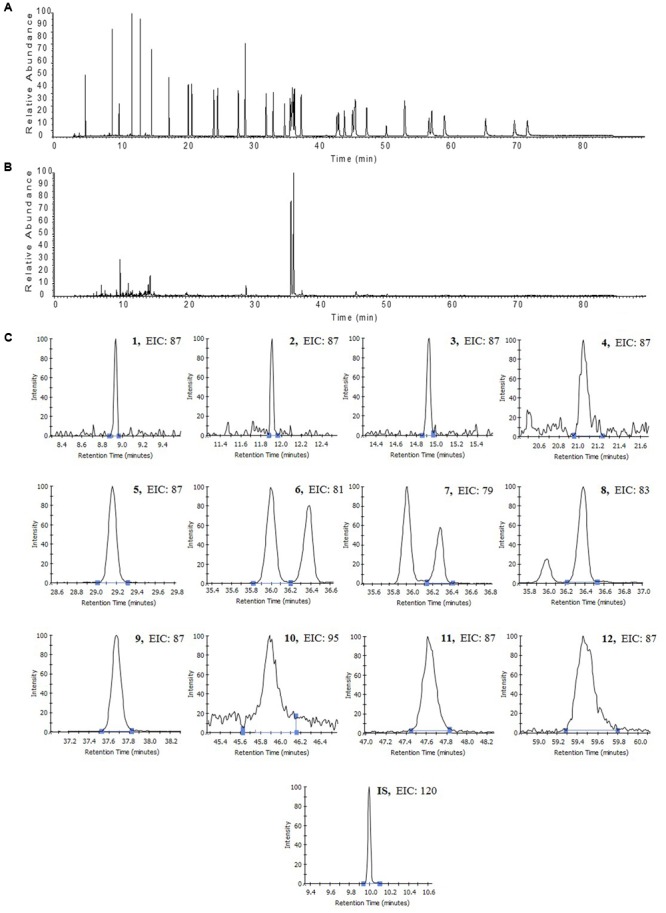
Total ion chromatography of mixed standards **(A)** and sample **(B)**, and EIC chromatography of sample **(C)** for fatty acids analyzed with gas chromatography coupled with mass spectrometry (GC-MS) method. Octanoic acid (1), decanoic acid (2), dodecanoic acid (3), tridecanoic acid (4), palmitic acid (5), linoleic acid (6), linolenic acid (7), oleinic acid (8), stearic acid (9), eicosenoic acid (10), eicosanoic acid (11), docosanoic acid (12), methyl salicylate (IS).

**FIGURE 6:**
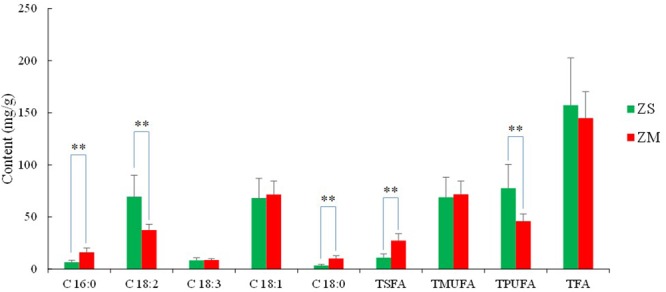
Contents of fatty acids (mg/g) in the seeds of *Z. jujuba* var. *spinosa* (ZS) and *Z. mauritiana* (ZM). Palmitic acid (C 16:0), linoleic acid (C 18:2), linolenic acid (C 18:3), oleinic acid (C 18:1), stearic acid (C 18:0); TSFA, TMUFA, TPUFA, and TTFA refer to the total content of SFA, MUFA, PUFA, and fatty acids, respectively. ^∗^*P* < 0.05, ^∗∗^*P* < 0.01.

Fatty acids were analyzed on a gas chromatography coupled with mass spectrometry (GC-MS) system after the sample being derivation, and the typical chromatograms of the standards and sample are presented in **Figure 5**. The validation results of the method are summarized in Supplementary Tables S8, S9, which showed that this GC-MS method could be suitable for determination of the fatty acids in the samples. Supplementary Table S10 lists the contents of individual fatty acids in the seeds of *Ziziphus* species. The results showed that 12 fatty acids including 8 saturated (SFA), 2 monounsaturated (MUFA), and 2 polyunsaturated (PUFA) fatty acids were found in both ZS and ZM samples, and no significant difference was found for the total contents of the fatty acids between the two samples of *Ziziphus* species. The major fatty acid in ZS samples found was linoleic acid which content ranged from 29.85 mg/g (ZS09) to 102.88 mg/g (ZS08), followed by oleic acid (32.31–93.96 mg/g) and linolenic acid (3.76–11.66 mg/g). Other fatty acids were found at a concentration of less than 10 mg/g. As for the ZM samples, the main fatty acid was found to be oleic acid (48.96–88.26 mg/g), followed by linoleic acid (29.78–46.58 mg/g), palmitic acid (11.60–20.48% mg/g), stearic acid (6.56–13.04 mg/g) and linolenic acid (6.79–10.95 mg/g). A significant difference (*P* < 0.01) between the two species was observed in the fatty acid profile (**Figure 6**). The ZS samples presented higher content of PUFA and lower content of SFA than the ZM samples. The average content of total unsaturated fatty acids in ZS samples was 146.37 mg/g which accounted for more than 90% of total fatty acids, while it was only 117.5 mg/g for ZM samples. For the PUFA, their average total content in ZS was 77.53 mg/g, while it was only 45.90 mg/g in ZM. On the contrary, the total level of SFA in ZS samples was far lower than that in ZM, and their average contents were 10.87 mg/g and 27.22 mg/g, respectively. It was reported that among the environmental factors considered, temperature plays an essential role in the fatty acid composition of seed oils, by regulating fatty acid desaturases (Luisa Hernandez et al., 2011; Borges et al., 2017). It has been shown that low temperatures increase the PUFA content of plants, thus maintaining the fluidity of biological membranes (Los and Murata, 1998). Corroborating this relationship, in the present study the ZS samples with high contents of PUFA were all collected in the northern area of China where the average temperature is relatively low, whereas the ZM samples were mainly distributed in Southeast Asia with the higher temperature and presented higher levels of SFA. Due to the high levels of the unsaturated fatty acids, ZS could be considered having higher potential health care value than ZM.

In addition, there were reports that the alkaloids contained in the ZS exhibited the sedative activity and contributed to the human health, and these compounds have used to quality control of the ZS samples (Park et al., 1991; Ma et al., 2008). Unfortunately, owing to the absence of the reference compounds, these alkaloids did not be used as indicators to evaluate the differences between these two *Ziziphus* seeds in present study, which should be investigated in the following studies.

### Multivariate PCA and OPLS-DA Analyses

The above analysis results revealed that the differences between ZS and ZM samples were found for various types of components. However, which components exhibited the greatest contribution for the difference and could be selected as the biomarkers for distinguishing the two species, was difficult to be found with the simple visual inspection. Thus, a more holistic investigation was performed with the multivariate PCA and OPLS-DA analyses to explore the relative variability within the samples for the two species.

**FIGURE 7:**
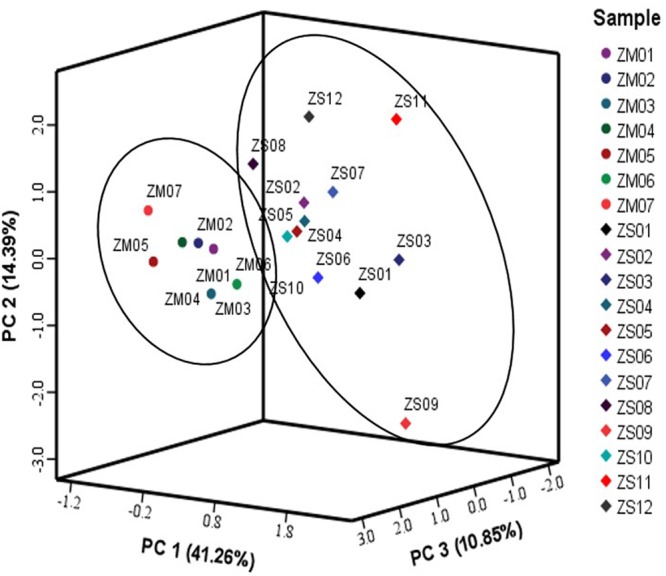
Score plot obtained by PCA of the seed samples from two *Ziziphus* species: *Z. jujuba* var. *spinosa* (ZS, ●) and *Z. mauritiana* (ZM, ♦).

**FIGURE 8:**
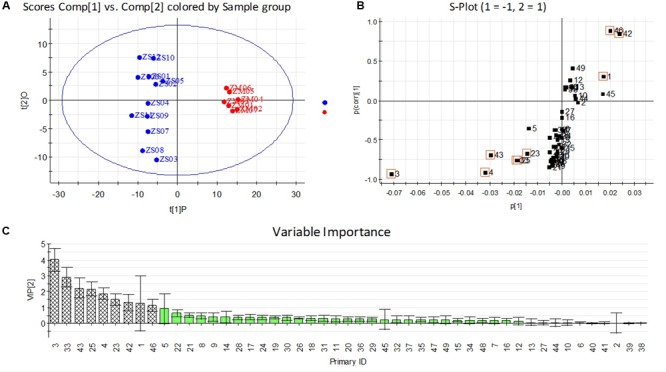
Orthogonal projections to latent structures-discriminant analysis (OPLS-DA) **(A)** score plot, **(B)** loading S-plots and **(C)** variable important plots obtained from two seeds of *Ziziphus* species: *Z. jujuba* var. *spinosa* (ZS, 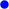) and *Z. mauritiana* (ZM, 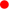) modeled separately. The S-plot shows the covariance *p*[1] against the correlation *p*(cor)[1] of the variables of the discriminating component of the OPLS-DA model. Cut-off values of *P* < 0.01 were used; variables are noted the number as Supplementary Table S8.

Principle component analysis is an unsupervised clustering method requiring no previous knowledge of the dataset. A total of 49 variables detected in the samples (5 variables of flavonoids and triterpenoids, 11 variables of nucleosides and nucleobases, 21 variables of amino acids and 12 variables of fatty acids), which were listed in Supplementary Table S11, were used in PCA analysis and the three major principle components (PC1, PC2, and PC3) accounting for 66.5% of the total variance were selected for construction of the score plot (**Figure 7**). As expected, PCA score plot showed a clear separation between the two kinds of samples, in which most of ZM samples were positioned at the left side of PC1 (negative side), while the ZS samples were located at the right side of PC1 (positive side). The observation confirmed generally that the differences did exist between the ZS and ZM samples. Consequently, a supervised OPLS-DA was carried out to enhance sample separation observed in the PCA model, as the latter has greater potential in the identification of markers by providing the most relevant variables for differentiation among sample groups (Khalil et al., 2017). The score plot from OPLS-DA analysis showed a clear separation between ZS and ZM samples (**Figure 8A**), which explained 97% of the total variance with the prediction goodness parameter *Q*^2^ = 0.93. The S-plot is a particularly useful tool that compares the variable magnitude against its reliability and is mainly used to filter out putative markers from “omics data,” in which the axes plotted from the predictive component are the covariance p[1] against the correlation *p*(cor) (El Senousy et al., 2014). Those variables located nearly the two ends of “S”’ in the S-plot represent those components contributing most to the difference between the samples with most confidence (Guo et al., 2011a). As shown in **Figure 8B**, most of the variable were located nearly the middle part of “S,” except the variables, such as 3, 4, 43, 33, 25, and 23 (which are located nearly the lower-left corner of “S”), and 46, 42, and 1 (which are located nearly the top-right corner of “S”). In addition, the above variables showed the high levels in the variable important plot (**Figure 8C**), suggesting that they contributed most of the difference between the seeds of two *Ziziphus* species. The results revealed that the ZS samples were generally more rich in saponins including jujuboside A (3) and jujuboside B (4), polyunsaturated fatty acid (linoleic acid, 43) and some amino acids, such as proline (23), tyrosine (25) and asparagine (33), whereas the ZM samples were particularly rich in saturated fatty acids and flavonoids, such as stearic acid (46), palmitic acid (42), and spinosin (1). Among them, jujubosides A and B could be used as the chemical markers for the discrimination between ZS and ZM samples due to their high contribution to the difference with most confidence, and if these compounds are absent, it may be a good indication for possible adulteration. These results are in agreement with the discovery in the content determination of these compounds, and are partially consistent with a previous reports (Zhang et al., 2016).

In fact, there was report that these two seeds could be differentiated just by their appearance including color and shape (Van der Valk et al., 2017), whereas this law was not universally applicable, since some samples collected from pharmacies had been pulverized or even parched. In addition, compared to the chromatographic fingerprinting and semi-quantitative analyses methods (Sun et al., 2014; Zhang et al., 2016), the approach presented here used the reference compounds for the analysis and could provide more accurate quantitative results for determining the chemical profiles’ difference between the two seeds of *Ziziphus* species.

## Conclusion

The methods for determination of total 59 analytes including 3 triterpenoids, 2 flavonoids, 19 nucleosides and nucleobases, 23 amino acids, and 12 fatty acids were established using HPLC-ELSD, ultra-high performance liquid chromatography coupled with triple-quadrupole mass spectrometry (UHPLC-TQ MS), and GC-MS, and total 49 compounds were detected in two seeds of *Ziziphus* species. The results revealed that the ZS samples were more rich in saponins, polyunsaturated fatty acids and some amino acids, whereas the ZM samples were particularly rich in saturated fatty acids and flavonoids, which suggested that the bioactivity of ZM for human health is not necessarily similar to that of ZS from their chemical profiles. These results would also be helpful for the choice of the suitable species due to the bioactivities and beneficial effects of these compounds for human health. For example, if taking saturated fatty acids and flavonoids as the major utilizing purpose, ZM should be the ideal choice. In contrast, ZS should be the best choice when taking saponins and polyunsaturated fatty acids as the major ones. In addition, although the chemical difference between these two seed of *Ziziphus* species was investigated, the mechanisms for the variation of these compound contents are not clear and need to be investigated in the following study.

## Author Contributions

SG, YL, and RW performed the experiments and analyzed the data. SG wrote the manuscript. HY, DQ, YT, and SS collected and prepared samples, revised the manuscript. J-AD designed the study and amended the paper. All authors have read and approved the final version.

## Conflict of Interest Statement

The authors declare that the research was conducted in the absence of any commercial or financial relationships that could be construed as a potential conflict of interest.
