# Supplementary material for: Comparison of the Bioactive Components in Two Seeds of Ziziphus Species by Different Analytical Approaches Combined with Chemometrics
Source: Front Pharmacol. 2017 Sep 5;8:609. doi: 10.3389/fphar.2017.00609 (PMC5591821; doi:10.3389/fphar.2017.00609)
Supplement: Supplementary file 1 [file Presentation_1.PDF]

Supplementary Material

Comparison of the bioactive components in two seeds of *Ziziphus* species by different analytical approaches coupled with chemometrics

Sheng Guo, Jin-ao Duan *, Yiqun Li, Ruiqing Wang, Hui Yan, Dawei Qian, Yuping Tang, Shulan Su

*** Correspondence:** Jin-ao Duan: dja@njucm.edu.cn

1 Supplementary Methods（*Validation of the method*）

The method was validated for linearity, limits of detection and quantification (LODs and LOQs), precision (inter-day and intra-day precision), repeatability, stability and accuracy following the International Conference on Harmonization (ICH) guideline.

1.1. Calibration curves, limits of detection and quantification

The working standard solutions with six different concentrations were analyzed, and the calibration curves were calculated by linear regression of the double logarithmic plots of the peak area versus the concentration of the reference solution injected for the determination of flavonoids and triterpenoids using HPLC-ELSD. For the determination of amino acids, nucleosides and fatty acids with UHPLC-TQ MS or GC-MS, calibration curves were constructed from peak areas of the reference standards versus their concentrations.

The limits of detection (LODs) and quantification (LOQs) for each analyte under the present chromatographic conditions were determined by diluting the standard solution when the signal-to-noise ratios (S/N) of analytes were about 3 and 10, respectively.

1.2. Precision, repeatability and stability

The precisions of the developed methods were evaluated with the intra-day and inter-day variations which were investigated by determining the analytes in six replicates during a single day and by duplicating the experiments on three consecutive days. Variations of the peak area were taken as the measures of precision. Repeatability was confirmed by analyzing the six independent analytical sample solutions prepared from the QC sample which were obtained by mixing the same weight of Z403, ZS04, ZS08 and ZS12. One of above sample solutions was stored at 20 °C and analyzed at 0, 2, 4, 8, 12, and 24 h, respectively, to evaluate the solution's stability. All these variations were expressed by relative standard deviation (RSD).

1.3 Accuracy

Recovery test was used to evaluate the accuracy of the methods. The tests were performed by adding accurate amounts of the standards into a certain amount (0.5 g) of QC sample separately. The spiked samples were then extracted, processed, and quantified according with the methods mentioned above. Six replicates were analyzed and the average recovery percentage was calculated by the formula: recovery (%) = (observed amount − original amount)/spiked amount × 100%.

2 Supplementary Figures

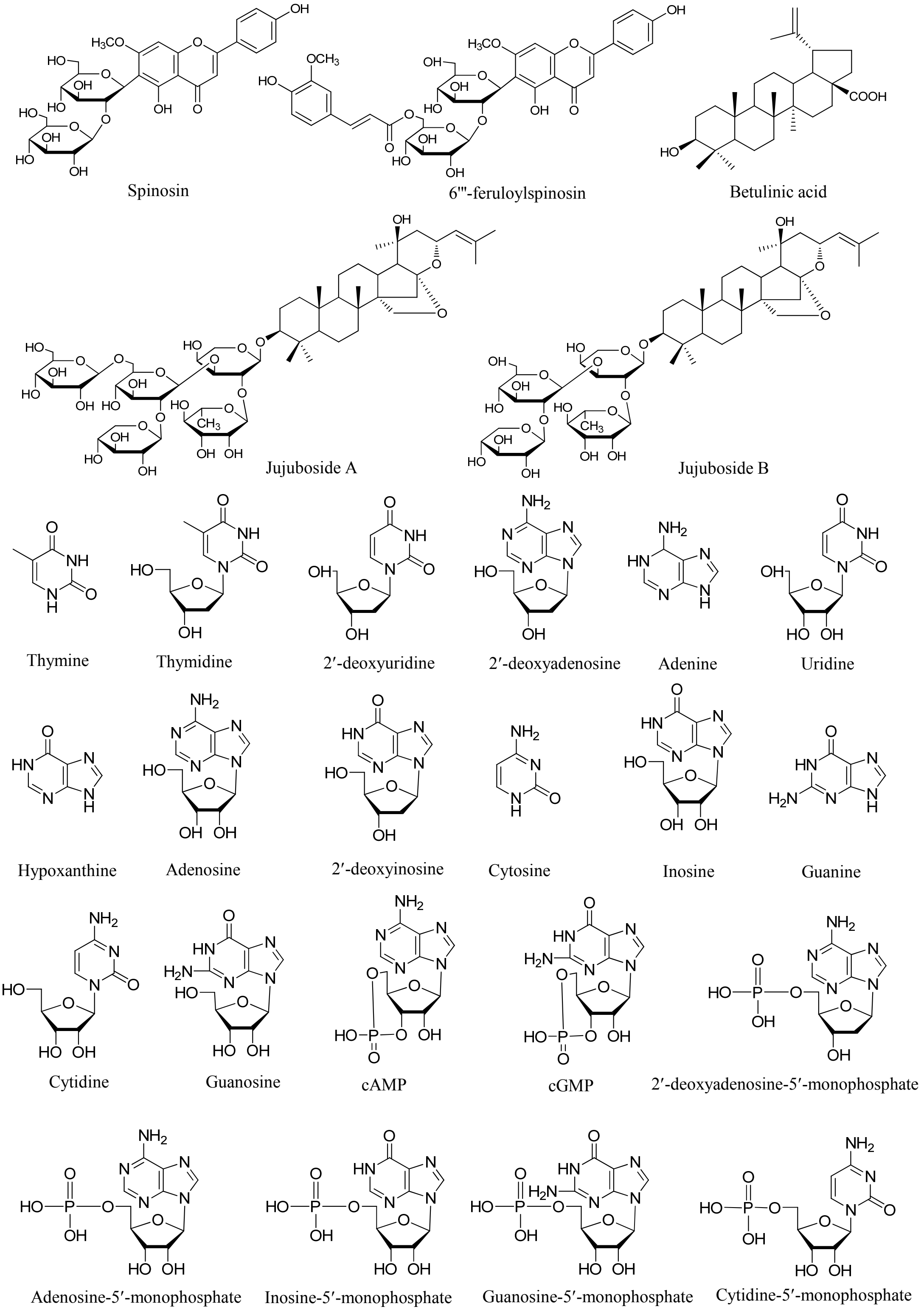

Figure S1. Structure of flavonoids, triterpenoids, nucleosides and nucleobases in the two seeds of *Ziziphus* species evaluated in the present study.

3 Supplementary Tables

Table S1. MS parameters of the investigated amino acids, nucleosides and nucleobases analyzed in this study

Analyte	Retention time (min)	ESI mode	MRM transitions/SIM	Cone voltage (V)	Collision energy (eV)
Thymine	1.40	ESI+	127.0	30	-
Thymidine	1.78	ESI+	243.1→127.0	10	10
2′-deoxyuridine	2.06	ESI+	229.0→112.9	8	10
2′-deoxyadenosine	2.83	ESI+	252.0→135.9	16	14
Adenine	2.83	ESI+	136.0	30	-
Uridine	3.31	ESI+	245.0→112.9	10	10
Hypoxanthine	3.75	ESI+	137.0	16	-
Adenosine	3.77	ESI+	268.0→135.9	22	18
2′-deoxyinosine	3.97	ESI+	253.0→136.9	22	12
Cytosine	5.27	ESI+	112.1→94.9	32	18
Inosine	5.80	ESI+	269.0→136.9	10	14
Guanine	7.20	ESI+	152.0	30	-
Cytidine	7.25	ESI+	243.9→111.9	28	10
Guanosine	7.25	ESI+	284.1→152.0	14	14
Phenylalanine	9.51	ESI+	166.1→120.0	18	14
Leucine	9.57	ESI+	132.1→86.1	16	10
Tryptophan	9.83	ESI+	205.1→146.0	16	18
Isoleucine	10.03	ESI+	132.1→86.1	16	10
Cysteine	10.59	ESI+	122.0→75.9	14	17
Methionine	10.61	ESI+	150.1→104.0	14	10
Taurine	10.77	ESI+	126.0→44.0	24	14
Proline	11.05	ESI+	116.0→70.0	20	10
Valine	11.20	ESI+	118.0→72.1	12	10
Tyrosine	11.93	ESI+	182.1→136.0	16	16
γ-aminobutyric acid	13.17	ESI+	103.9→87.0	12	6
Hydroxyproline	14.19	ESI+	132.0→67.9	18	16
Alanine	14.26	ESI+	90.0→44.0	16	10
Threonine	15.51	ESI+	120.0→14.0	38	20
Lysine	16.60	ESI+	147.0→56.0	14	14
Glutamine	16.62	ESI+	147.0→83.9	8	16
Serine	16.63	ESI+	106.0→60.0	14	8
Asparagine	16.69	ESI+	132.9→73.9	12	14
Citrulline	16.8	ESI+	176.0→69.9	16	20
2′-deoxyadenosine-5′-monophosphate	16.84	ESI+	332.0→135.9	20	16
Glutamic Acid	16.86	ESI+	147.9→83.9	12	14
Adenosine-5′-monophosphate	16.94	ESI-	346.2→134.0	30	26
Inosine-5′-monophosphate	17.05	ESI+	349.2→137.0	14	12
Aspartic acid	17.06	ESI+	134.0→88.0	14	10
Guanosine-5′-monophosphate	17.24	ESI-	362.2→211.0	26	16
Cytidine-5′-monophosphate	17.27	ESI+	324.0→111.9	16	14
Histidine	17.28	ESI+	156.1→110.0	20	16
Arginine	17.3	ESI+	175.2→70.0	22	18
Ornithine	17.87	ESI+	133.0→69.9	14	14

Table S2. MS parameters of the investigated fatty acids analyzed in this study

Analyte	Retention time (min)	Quantitative ion (*m*/*z*)	Monitoring ion (*m*/*z*)		
Octanoic acid	8.93	87	74	87	127
Decanoic acid	11.91	87	74	87	143
Dodecanoic acid	14.91	87	74	87	143
Tridecanoic acid	21.05	87	74	87	143
Palmitic acid	29.18	87	74	87	143
Linoleic acid	36.00	81	67	81	95
Linolenic acid	36.20	79	67	79	95
Oleinic acid	36.37	83	55	83	97
Stearic acid	37.67	87	74	87	143
Eicosenoic acid	45.92	95	69	79	95
Eicosanoic acid	47.63	87	74	87	143
Docosanoic acid	59.48	87	74	87	143

Table S3. Regression equation, correlation coefficients, linearity ranges and limit of detection (LOD) and quantitation (LOQ) of the investigated flavonoids and triterpenoids.

Analyte	Calibration curves [a]	r^2	Linear range (μg)	LOD (ng)	LOQ (ng)
Spinosin	y = 1.2912x + 1.1699	0.9989	0.136~5.440	30	34
6'''-feruloylspinosin	y = 1.3869x + 0.9172	0.9996	0.086~3.424	22	43
Jujuboside A	y = 1.3823x + 0.4654	0.9984	0.118~4.704	28	59
Jujuboside B	y = 0.9641x + 1.7825	0.9991	0.076~3.056	35	76
Betulinic acid	y = 1.7085x - 0.9122	0.9996	0.125~2.496	30	62

[a] y is the logarithmic value of peak area, and x is the logarithmic value of reference compound's amount (μg).

Table S4. Precision, repeatability, stability and recovery of the investigated flavonoids and triterpenoids.

Analyte	Precision (RSD, %)		Repeatability (RSD, %, n=6)	Stability (RSD, %, n=6)	Recovery (%, n=3)	
	Intra-day (n=6)	Inter-day (n=3)			Mean	RSD,%
Spinosin	1.11	2.02	2.58	1.93	97.0	2.5
6'''-feruloylspinosin	1.28	2.33	2.18	2.00	98.3	1.9
Jujuboside A	3.09	2.14	3.36	2.87	97.5	2.1
Jujuboside B	1.87	3.02	2.99	2.61	98.4	2.4
Betulinic acid	1.74	2.54	2.61	2.53	101.2	2.9

Table S5. Validation of the method for determination of amino acids, nucleosides and nucleobases

Analyte	Calibration curves [a]	Linear range (μg/ml)	r^2	Precision (RSD, %)		Repeatability (RSD, %, n=6)	Stability (RSD, %, n=6)	Recovery (%, n=3)	
				Intra-day (n=6)	Intra-day (n=6)			Mean	RSD,%
Thymine	$y = 6.7\times10^4x + 6.8\times10^2$	0.052~52.2	0.9982	2.23	3.12	3.55	3.14	97.2	3.55
Thymidine	$y = 6.9\times10^5x + 3.1\times10^2$	0.048~48.4	0.9990	2.89	2.59	-	-	95.8	2.98
2′-deoxyuridine	$y = 6.0\times10^4x + 1.0\times10^2$	0.049~49.3	0.9993	2.14	3.02	-	-	98.1	3.52
2′-deoxyadenosine	$y = 2.3\times10^7x – 2.5\times10^4$	0.006~6.20	0.9991	2.05	3.11	3.82	3.08	97.6	4.10
Adenine	$y = 3.8\times10^7x – 3.1\times10^4$	0.044~44.1	0.9991	2.64	3.45	3.21	3.54	96.1	4.08
Uridine	$y = 3.3\times10^5x + 3.8\times10^2$	0.057~56.9	0.9990	2.64	2.91	3.38	4.02	95.8	3.64
Hypoxanthine	$y = 6.9\times10^6x + 5.8\times10^2$	0.042~41.8	0.9981	2.00	2.66	2.97	3.08	102.3	3.07
Adenosine	$y = 1.5\times10^8x – 1.6\times10^5$	0.041~41.0	0.9987	2.12	3.56	2.97	3.00	97.3	3.47
2′-deoxyinosine	$y = 1.3\times10^7x – 5.3\times10^4$	0.098~98.1	0.9991	2.21	3.71	-	-	103.0	3.08
Cytosine	$y = 4.4\times10^7x + 1.2\times10^3$	0.005~5.17	0.9985	3.13	2.55	3.52	3.29	99.1	3.23
Inosine	$y = 8.1\times10^6x – 2.1\times10^3$	0.003~3.49	0.9989	2.52	3.61	3.02	2.97	98.0	3.12
Guanine	$y = 2.5\times10^6x – 1.8\times10^3$	0.025~25.0	0.9980	2.08	2.46	-	-	99.0	3.08
Cytidine	$y = 8.5\times10^5x – 2.5\times10^2$	0.004~3.84	0.9971	2.52	3.89	2.39	3.52	96.7	2.94
Guanosine	$y = 5.2\times10^6x – 2.4\times10^3$	0.052~52.0	0.9969	3.20	4.02	3.08	4.21	98.2	3.41
Phenylalanine	$y = 1.2\times10^7x – 1.5\times10^4$	0.601~60.6	0.9952	2.13	3.91	3.03	2.64	96.1	3.82
Leucine	$y = 6.8\times10^6x – 9.5\times10^3$	0.044~44.1	0.9982	2.67	2.97	2.98	3.11	97.3	3.52
Tryptophan	$y = 2.7\times10^6x – 7.8\times10^3$	0.053~52.8	0.9958	2.82	3.62	2.97	3.58	95.5	3.28
Isoleucine	$y = 4.7\times10^6x – 2.6\times10^2$	0.031~30.7	0.9990	1.87	3.14	3.24	4.05	98.5	2.57
Cysteine	$y = 6.8\times10^4x + 58$	0.121~242	0.9991	2.34	4.03	3.33	4.17	95.1	4.52
Methionine	$y = 3.0\times10^5x – 7.7\times10^2$	0.051~50.6	0.9990	2.53	3.53	2.89	3.41	99.59	2.93
Taurine	$y = 2.8\times10^4x + 97$	0.082~82.4	0.9979	2.57	3.14	-	-	98.9	2.21
Proline	$y = 3.2\times10^5x + 1.6\times10^2$	0.091~91.4	0.9963	2.36	2.82	2.94	3.25	97.0	3.18
Valine	$y = 5.6\times10^5x – 1.6\times10^3$	0.082~82.0	0.9983	2.67	4.33	3.54	4.05	96.4	3.51
Tyrosine	$y = 1.3\times10^5x + 50$	0.048~48.2	0.9991	3.13	4.82	3.83	3.88	95.8	2.82
γ-aminobutyric acid	$y = 4.0\times10^5x + 2.2\times10^2$	0.036~35.7	0.9990	1.99	3.99	3.18	4.57	103.2	3.55
Hydroxyproline	$y = 1.3\times10^5x – 3.0\times10^2$	0.052~52.0	0.9998	3.20	4.08	3.99	2.98	97.0	2.60
Alanine	$y = 7.7\times10^4x + 2.2\times10^2$	0.087~86.6	0.9978	1.74	3.26	4.03	3.58	96.2	3.45
Threonine	$y = 6.8\times10^5x + 95$	0.064~64.3	0.9968	2.60	3.77	2.79	4.14	101.3	3.29
Lysine	$y = 1.4\times10^5x – 1.8\times10^2$	0.055~55.4	0.9988	2.27	3.09	3.87	3.68	96.9	4.12
Glutamine	$y = 2.6\times10^5x – 4.0\times10^2$	0.062~62.1	0.9989	2.97	3.61	3.08	2.97	95.8	2.99
Serine	$y = 2.3\times10^5x + 44$	0.054~54.1	0.9969	3.19	3.22	3.89	4.12	98.2	3.05
Asparagine	$y = 3.0\times10^4x - 0.88$	0.049~49.4	0.9975	3.02	2.97	3.36	3.02	96.2	3.85
Citrulline	$y = 3.2\times10^5x – 6.5\times10^2$	0.055~54.6	0.9972	2.58	4.01	2.99	2.57	97.1	3.15
2′-deoxyadenosine-5′-monophosphate	$y = 1.8\times10^5x – 2.8\times10^2$	0.044~44.3	0.9983	2.99	3.35	-	-	98.3	3.02
Glutamic Acid	$y = 5.7\times10^4x - 4$	0.061~60.9	0.9990	2.90	3.66	3.10	4.05	96.7	3.62
Adenosine-5′-monophosphate	$y = 6.2\times10^4x + 46$	0.038~38.2	0.9992	2.73	3.03	-	-	97.2	2.64
Inosine-5′-monophosphate	$y = 9.8\times10^5x + 15$	0.036~35.9	0.9990	2.90	4.25	-	-	98.3	2.59
Aspartic acid	$y = 1.1\times10^4x + 10$	0.068~68.2	0.9984	2.97	4.38	-	-	103.1	3.09
Guanosine-5′-monophosphate	$y = 6.8\times10^4x - 17$	0.034~34.0	0.9986	2.55	3.55	3.87	3.36	96.3	3.42
Cytidine-5′-monophosphate	$y = 4.8\times10^4x - 53$	0.082~82.1	0.9988	3.30	3.91	-	-	96.2	2.96
Histidine	$y = 8.1\times10^5x – 1.5\times10^3$	0.055~55.3	0.9975	2.23	3.46	3.55	4.19	97.2	3.13
Arginine	$y = 7.0\times10^5x – 8.8\times10^2$	0.049~49.3	0.9988	2.99	3.85	3.08	3.82	98.3	3.97
Ornithine	$y = 1.1\times10^5x – 3.1\times10^2$	0.038~38.0	0.9987	1.85	3.07	3.08	2.85	96.9	3.54

Table S6. The profiles of free amino acids (mg/100 g) in the seeds of *Z. jujuba* var. *spinosa* and *Z. mauritiana* (mean ± SD).

Analytes	Seeds of *Ziziphus jujuba* var. *spinosa*													Seeds of *Ziziphus mauritiana*							
	ZS01	ZS02	ZS03	ZS04	ZS05	ZS06	ZS07	ZS08	ZS09	ZS10	ZS11	ZS12	mean	ZM01	ZM02	ZM03	ZM04	ZM05	ZM06	ZM07	mean
Phenylalanine	7.61 ± 0.27^{c}	4.52 ± 0.13^{b}	19.06 ± 0.51^{f}	6.58 ± 0.20^{c}	7.80 ± 0.32^{c}	5.13 ± 0.15^{b}	11.08 ± 0.33^{d}	12.34 ± 0.63^{d}	27.03 ± 1.16^{g}	2.42 ± 0.06^{a}	13.90 ± 0.49^{e}	4.16 ± 0.14^{b}	10.14 ± 7.14	3.64 ± 0.13^{d}	1.82 ± 0.08^{c}	4.19 ± 0.12^{d}	0.44 ± 0.02^{a}	0.54 ± 0.02^{a}	1.20 ± 0.04^{b}	1.72 ±0.05^{c}	1.94 ± 1.46
Leucine	10.61 ± 0.34^{d}	5.72 ± 0.15^{b}	28.51 ± 0.89^{g}	8.69 ± 0.36^{c}	7.92 ± 0.25^{c}	5.41 ± 0.16^{b}	11.72 ± 0.36^{d}	14.88 ± 0.40^{e}	36.80 ± 1.48^{h}	2.63 ± 0.09^{a}	21.21 ± 0.68^{f}	4.24 ± 0.10^{ab}	13.20 ± 10.55	3.63 ± 0.11^{d}	1.48 ± 0.04^{c}	4.52 ± 0.23^{e}	0.31 ± 0.01^{a}	0.42 ± 0.01^{a}	0.81 ± 0.03^{b}	1.12 ± 0.06^{b}	1.76 ± 1.65
Tryptophan	3.61 ± 0.11^{d}	2.23 ± 0.08^{b}	7.20 ± 0.23^{h}	2.71 ± 0.10^{c}	3.59 ± 0.15^{d}	1.87 ± 0.07^{b}	5.91 ± 0.16^{f}	6.42 ± 0.20^{g}	9.73 ± 0.34^{i}	1.32 ± 0.04^{a}	5.22 ± 0.12^{e}	2.01 ± 0.07^{b}	4.32 ± 2.59	2.14 ± 0.07^{d}	1.17 ± 0.04^{bc}	1.32 ± 0.05^{c}	0.34 ± 0.01^{a}	0.75 ± 0.02^{b}	1.86 ± 0.06^{d}	1.24 ± 0.04^{c}	1.26 ± 0.61
Isoleucine	6.93 ± 0.19^{d}	4.40 ± 0.18^{b}	19.52 ± 0.60i	6.88 ± 0.18^{d}	5.84 ± 0.21^{c}	3.93 ± 0.12^{b}	7.91 ± 0.33^{e}	11.08 ± 0.33^{f}	18.32 ± 0.60^{h}	1.83 ± 0.05^{a}	12.16 ± 0.27^{g}	3.71 ± 0.11^{b}	8.54 ± 5.69	2.24 ± 0.09^{d}	1.22 ± 0.04^{b}	1.69 ± 0.06^{c}	0.64 ± 0.02^{a}	0.52 ± 0.01^{a}	1.21 ± 0.05^{b}	1.33 ± 0.06^{b}	1.26 ± 0.59
Methionine	3.45 ± 0.15^{e}	2.55 ± 0.08^{cd}	6.67 ± 0.33^{g}	3.64 ± 0.13^{e}	2.02 ± 0.09^{bc}	1.89 ± 0.06^{b}	2.89 ± 0.15^{d}	2.36 ± 0.11^{bcd}	6.94 ± 0.37^{g}	0.32 ± 0.01^{a}	4.93 ± 0.28^{f}	0.74 ± 0.03^{a}	3.20 ± 2.09	1.01 ± 0.04^{b}	nd	0.74 ± 0.04^{a}	nd	nd	nd	0.54 ± 0.03^{a}	0.33 ± 0.43
Proline	14.42 ± 0.61^{c}	8.89 ± 0.33^{b}	33.22 ± 1.03^{h}	20.83 ± 1.07^{e}	17.44 ± 0.52^{d}	7.22 ± 0.24^{b}	15.44 ± 0.47^{cd}	13.56 ± 0.56^{c}	30.54 ± 1.22^{g}	2.44 ± 0.10^{a}	25.89 ± 0.75^{f}	4.42 ± 0.11^{a}	16.19 ± 9.93	2.91 ± 0.12^{c}	1.89 ± 0.08^{b}	1.78 ± 0.07^{b}	0.63 ± 0.02^{a}	0.84 ± 0.04^{a}	1.64 ± 0.07^{b}	nd	1.38 ± 0.96
Valine	10.41 ± 0.37^{d}	7.01 ± 0.21b^{c}	28.33 ± 1.28^{g}	10.45 ± 0.38^{d}	8.23 ± 0.41^{c}	6.24 ± 0.23^{bc}	10.78 ± 0.37^{d}	15.54 ± 0.50^{e}	31.03 ± 1.59^{h}	2.64 ± 0.08^{a}	18.12 ± 0.78^{f}	5.22 ± 0.26^{b}	12.83 ± 8.95	4.44 ± 0.21^{d}	1.89 ± 0.07^{b}	3.11 ± 0.13^{c}	0.43 ± 0.01^{a}	0.32 ± 0.01^{a}	1.67 ± 0.05^{b}	1.54 ± 0.07^{b}	1.91 ± 1.46
Tyrosine	19.22 ± 0.70^{bc}	20.21 ± 0.63^{c}	44.44 ± 1.79^{f}	30.32 ± 1.31^{d}	19.80 ± 0.59^{c}	18.31 ± 0.43^{bc}	38.42 ± 1.54^{e}	46.45 ± 1.64^{f}	62.43 ± 1.86^{g}	13.42 ± 0.49^{a}	34.89 ± 1.39^{e}	16.22 ± 0.73^{ab}	30.34 ± 15.20	6.54 ± 0.26^{d}	4.78 ± 0.20^{c}	3.54 ± 0.15^{b}	nd	1.73 ± 0.07^{a}	nd	nd	2.37 ± 2.64
γ-aminobutyric acid	6.82 ± 0.21^{c}	6.93 ± 0.22^{c}	40.45 ± 1.45^{h}	20.70 ± 0.62^{e}	4.12 ± 0.14^{b}	6.63 ± 0.33^{c}	9.42 ± 0.40^{d}	26.11 ± 1.31^{g}	11.22 ± 0.40^{d}	1.14 ± 0.05^{a}	23.82 ± 0.73^{f}	5.34 ± 0.15^{bc}	13.56 ± 11.71	1.36 ± 0.05^{c}	0.66 ± 0.02^{b}	0.68 ± 0.02^{b}	nd	0.12 ± 0.00^{a}	0.24 ± 0.01^{a}	1.20 ± 0.06^{c}	0.61 ± 0.53
Hydroxyproline	nd	nd	nd	0.60 ± 0.02^{a}	nd	nd	nd	nd	2.80 ± 0.14^{b}	nd	nd	nd	0.28 ± 0.81	0.70 ± 0.03	nd	nd	nd	nd	nd	nd	0.10 ± 0.26
Alanine	19.89 ± 0.59d^{e}	16.54 ± 0.60^{c}	46.81 ± 2.35^{i}	22.11 ± 0.96^{ef}	9.67 ± 0.37^{b}	17.67 ± 0.70^{cd}	16.12 ± 0.65^{c}	23.03 ± 0.82^{f}	34.33 ± 1.45^{h}	5.53 ± 0.27^{a}	26.14 ± 1.01^{g}	5.11 ± 0.21^{a}	20.25 ± 11.85	3.67 ± 0.13^{b}	2.14 ± 0.08^{a}	7.40 ± 0.30^{c}	1.78 ± 0.09^{a}	3.04 ± 0.16^{b}	3.01 ± 0.14^{b}	nd	3.01 ± 2.27
Threonine	13.45 ± 0.45^{e}	11.41 ± 0.44^{d}	36.50 ± 1.26^{g}	8.02 ± 0.32^{bc}	35.60 ± 1.11^{g}	8.54 ± 0.26^{bc}	7.50 ± 0.29^{b}	8.21 ± 0.29^{bc}	26.53 ± 0.77^{f}	0.74 ± 0.04^{a}	9.63 ± 0.35^{c}	1.78 ± 0.07^{a}	13.99 ± 12.14	2.73 ± 0.11^{b}	1.34 ± 0.05^{a}	2.78 ± 0.11^{b}	nd	nd	nd	nd	0.98 ± 1.31
Lysine	16.22 ± 0.47^{d}	11.70 ± 0.41b^{cd}	29.00 ± 1.45^{f}	13.72 ± 0.58^{cd}	11.67 ± 0.50b^{cd}	21.24 ± 0.78^{e}	7.41 ± 0.24^{ab}	30.43 ± 1.23^{f}	115.78 ± 5.23^{g}	9.33 ± 0.39abc	16.22 ± 0.64^{d}	4.94 ± 0.18^{a}	23.97 ± 29.97	2.45 ± 0.10^{a}	6.10 ± 0.26^{c}	4.78 ± 0.20^{b}	nd	nd	nd	2.80 ± 0.12^{a}	2.30 ± 2.47
Glutamine	15.51 ± 0.60^{ef}	8.23 ± 0.25^{cd}	31.52 ± 1.25^{h}	9.78 ± 0.29^{d}	8.01 ± 0.33^{cd}	21.45 ± 0.78^{g}	7.32 ± 0.27^{bc}	16.00 ± 0.52ef	42.45 ± 1.71i	5.56 ± 0.19ab	12.67 ± 0.52^{e}	4.41 ± 0.16^{a}	15.24 ± 11.51	3.70 ± 0.14^{b}	3.04 ± 0.12^{b}	9.00 ± 0.36^{d}	nd	nd	1.11 ± 0.03^{a}	4.72 ± 0.20^{c}	3.08 ± 3.19
Serine	14.80 ± 0.61b^{cd}	17.11 ± 0.61^{cd}	55.54 ± 2.50^{f}	20.34 ± 0.60^{d}	9.41 ± 0.41^{ab}	12.42 ± 0.50^{bc}	90.89 ± 3.33^{g}	134.14 ± 5.38^{h}	43.92 ± 1.70^{e}	5.67 ± 0.18^{a}	39.45 ± 1.57^{e}	15.00 ± 0.68^{bcd}	38.22 ± 39.98	13.20 ± 0.48^{a}	15.81 ± 0.50^{a}	nd	nd	nd	nd	68.32 ± 3.08^{b}	13.90 ± 24.96
Asparagine	54.41 ± 1.93^{e}	28.54 ± 0.85^{c}	99.33 ± 3.64^{h}	45.42 ± 1.90^{d}	19.51 ± 0.98^{b}	73.11 ± 2.18^{g}	72.28 ± 3.06^{fg}	92.43 ± 4.64^{h}	65.27 ± 3.22^{f}	24.19 ± 0.86^{bc}	57.44 ± 2.41^{e}	12.01 ± 0.61^{a}	53.66 ± 28.50	7.89 ± 0.31^{a}	nd	13.59 ± 0.56^{b}	nd	nd	nd	nd	3.07 ± 5.49
Citrulline	1.58 ± 0.07^{c}	nd	nd	1.22 ± 0.05^{b}	nd	nd	nd	nd	nd	nd	2.47 ± 0.11^{d}	1.09 ± 0.05^{a}	0.53 ± 0.85	nd	nd	nd	nd	nd	nd	nd	–
Glutamic Acid	36.33 ± 1.49^{e}	10.22 ± 0.36^{bc}	59.32 ± 2.27^{h}	16.67 ± 0.52^{d}	15.94 ± 0.48^{d}	11.73 ± 0.48^{c}	18.89 ± 0.78^{d}	53.21 ± 2.67^{g}	49.20 ± 1.79^{f}	7.62 ± 0.25^{ab}	34.22 ± 1.32^{e}	5.31 ± 0.24^{a}	26.56 ± 19.11	10.89 ± 0.50^{b}	10.44 ± 0.38^{b}	9.11 ± 0.32^{b}	2.67 ± 0.08^{a}	2.72 ± 0.08^{a}	11.20 ± 0.36^{b}	1.89 ± 0.08^{a}	6.99 ± 4.33
Histidine	9.26 ± 0.41^{cd}	7.44 ± 0.26^{bc}	29.89 ± 1.19^{i}	15.11 ± 0.44^{ef}	10.37 ± 0.43^{d}	10.56 ± 0.34^{d}	14.41 ± 0.50^{e}	23.34 ± 1.02^{g}	25.67 ± 1.05^{h}	4.48 ± 0.17^{a}	16.56 ± 0.61^{f}	5.82 ± 0.29^{ab}	14.41 ± 8.15	9.04 ± 0.36^{e}	4.63 ± 0.19^{c}	6.20 ± 0.18^{d}	1.80 ± 0.07^{b}	0.93 ± 0.03^{a}	2.44 ± 0.10^{b}	6.37 ± 0.23^{d}	4.49 ± 2.92
Arginine	30.91 ± 1.12^{ab}	23.18 ± 0.64^{a}	131.62 ± 4.82^{f}	73.78 ± 2.34^{e}	54.38 ± 2.57^{d}	52.22 ± 1.84^{cd}	121.37 ± 4.96^{f}	166.67 ± 6.68^{g}	283.41 ± 12.27^{h}	38.70 ± 1.76^{bc}	153.59 ± 5.42^{g}	33.91 ± 1.35^{ab}	96.98 ± 77.55	47.12 ± 1.82^{c}	16.66 ± 0.65^{b}	47.45 ± 2.16^{c}	7.90 ± 0.31^{a}	10.44 ± 0.32^{a}	50.14 ± 2.32^{c}	45.28 ± 1.74^{c}	32.14 ± 19.38
Ornithine	nd	nd	2.23 ± 0.11^{c}	nd	nd	1.74 ± 0.07^{b}	nd	nd	nd	nd	0.80 ± 0.04^{a}	4.89 ± 0.24^{d}	0.81 ± 1.50	nd	nd	nd	nd	nd	nd	nd	–
Total amino acids	295.44 ± 10.66^{cd}	196.83 ± 6.44^{b}	749.16 ± 28.92^{g}	337.57 ± 12.36^{d}	251.32 ± 9.84^{bc}	287.31 ± 9.73^{cd}	469.76 ± 18.19^{e}	696.20 ± 28.91^{g}	923.40 ± 38.36^{h}	129.98 ± 5.08^{a}	529.33 ± 19.48^{f}	140.33 ± 5.80^{a}	417.22 ± 258.12	129.30 ± 5.08^{c}	75.07 ± 2.80^{b}	121.88 ± 5.07^{c}	16.94 ± 0.63^{a}	22.37 ± 0.79^{a}	76.53 ± 3.27^{b}	138.07 ± 5.81^{c}	82.88 ± 49.72
Total essential amino acids	72.29 ± 2.32^{de}	49.54 ± 1.68^{b}	174.79 ± 6.55^{g}	60.69 ± 2.26^{bcd}	82.67 ± 3.04^{e}	54.25 ± 1.83^{bc}	65.20 ± 2.23^{cd}	101.26 ± 3.68^{f}	272.16 ± 11.54^{h}	21.23 ± 0.77^{a}	101.39 ± 3.68^{f}	26.80 ± 0.98^{a}	90.19 ± 70.13	22.28 ± 0.86^{e}	15.02 ± 0.58^{d}	23.13 ± 0.95^{e}	2.16 ± 0.07^{a}	2.55 ± 0.08^{a}	6.75 ± 0.23^{b}	10.29 ± 0.42^{c}	11.74 ± 8.71

Sample no. is same as Table1; Means within a |ine with different superscripts differ significantly ($P < 0.05$); nd = not detected. Cysteine, taurine and aspartic acid were not detected in all the samples.

Table S7. The profiles of nucleosides and nucleobases (mg/100 g) in the seeds of *Z. jujuba* var. *spinosa* and *Z. mauritiana* (mean ± SD).

Analytes	Seeds of *Ziziphus jujuba* var. *spinosa*													Seeds of *Ziziphus mauritiana*							
	ZS01	ZS02	ZS03	ZS04	ZS05	ZS06	ZS07	ZS08	ZS09	ZS10	ZS11	ZS12	mean	ZM01	ZM02	ZM03	ZM04	ZM05	ZM06	ZM07	mean
Thymine	0.17 ± 0.01^{b}	nd	0.14 ± 0.00^{a}	nd	nd	nd	nd	nd	nd	nd	0.22 ± 0.01^{c}	nd	0.04 ± 0.08	nd	nd	nd	nd	nd	nd	nd	–
2′-deoxyadenosine	0.26 ± 0.01^{f}	0.05 ± 0.00^{c}	0.25 ± 0.01^{f}	0.07 ± 0.00^{d}	0.03 ± 0.00^{b}	0.01 ± 0.00^{a}	0.06 ± 0.00^{cd}	0.05 ± 0.00^{cd}	0.13 ± 0.01^{e}	0.01 ± 0.00^{a}	0.33 ± 0.01^{g}	0.02 ± 0.00^{ab}	0.11 ± 0.11	0.02 ± 0.00^{a}	0.01 ± 0.00^{a}	0.09 ± 0.00^{c}	nd	nd	0.07 ± 0.00^{b}	0.12 ± 0.01^{d}	0.05 ± 0.05
Adenine	2.95 ± 0.08^{d}	1.42 ± 0.04^{b}	5.07 ± 0.15^{g}	2.17 ± 0.06^{c}	2.28 ± 0.08^{c}	1.33 ± 0.05^{b}	4.48 ± 0.13^{f}	2.80 ± 0.10^{d}	2.82 ± 0.11^{d}	0.63 ± 0.02^{a}	3.68 ± 0.10^{e}	1.27 ± 0.04^{b}	2.58 ± 1.35	0.80 ± 0.04^{cd}	0.54 ± 0.02^{b}	0.69 ± 0.04^{c}	0.27 ± 0.01^{a}	0.50 ± 0.02^{b}	2.56 ± 0.09^{e}	0.90 ± 0.02^{d}	0.89 ± 0.71
Uridine	3.22 ± 0.11^{e}	1.41 ± 0.04^{b}	7.25 ± 0.17^{i}	2.93 ± 0.11d^{e}	3.92 ± 0.15^{f}	0.65 ± 0.03^{a}	3.88 ± 0.10^{f}	5.24 ± 0.18^{g}	2.60 ± 0.07^{d}	1.77 ± 0.07^{c}	6.12 ± 0.18^{h}	1.69 ± 0.06^{bc}	3.39 ± 2.00	2.60 ± 0.09^{e}	1.77 ± 0.07^{c}	2.28 ± 0.11^{d}	nd	0.69 ± 0.03^{a}	2.75 ± 0.09e	1.27 ± 0.05b	1.62 ± 0.95
Hypoxanthine	0.34 ± 0.01^{de}	0.11 ± 0.01^{a}	0.58 ± 0.02^{g}	0.18 ± 0.01^{ab}	0.23 ± 0.01^{bc}	0.15 ± 0.01^{ab}	0.28 ± 0.01^{cd}	2.75 ± 0.09^{h}	0.45 ± 0.02^{f}	0.11 ± 0.01^{a}	0.41 ± 0.02^{ef}	0.16 ± 0.01^{ab}	0.48 ± 0.73	0.79 ± 0.03^{c}	0.36 ± 0.01^{b}	4.25 ± 0.11^{d}	nd	0.07 ± 0.00^{a}	0.70 ± 0.03^{c}	0.78 ± 0.03^{c}	0.99 ± 1.36
Adenosine	3.02 ± 0.12^{d}	2.77 ± 0.10^{d}	6.49 ± 0.15^{g}	2.74 ± 0.10^{d}	2.76 ± 0.11^{d}	1.59 ± 0.08^{b}	9.20 ± 0.22^{h}	3.82 ± 0.12^{f}	3.44 ± 0.13^{e}	1.07 ± 0.04^{a}	3.63 ± 0.10^{ef}	2.38 ± 0.07^{c}	3.58 ± 2.22	2.32 ± 0.08^{f}	1.78 ± 0.06^{e}	1.53 ± 0.06^{d}	0.78 ± 0.04c	0.39 ± 0.02b	0.04 ± 0.00^{a}	1.32 ± 0.06^{d}	1.16 ± 0.75
Cytosine	0.01 ± 0.00^{b}	0.01 ± 0.00^{b}	0.03 ± 0.00^{e}	0.01 ± 0.00^{a}	nd	0.01 ± 0.00^{b}	nd	0.01 ± 0.00^{c}	nd	nd	0.02 ± 0.00^{d}	nd	0.01 ± 0.01	0.02 ± 0.00^{b}	0.02 ± 0.00^{b}	nd	nd	nd	0.01 ± 0.00^{a}	0.12 ± 0.01^{c}	0.02 ± 0.04
Inosine	nd	0.05 ± 0.00^{bc}	nd	nd	0.06 ± 0.00^{cd}	0.02 ± 0.00^{abc}	nd	1.05 ± 0.04^{g}	0.31 ± 0.01^{f}	0.20 ± 0.01^{e}	0.02 ± 0.00^{ab}	0.09 ± 0.00^{d}	0.15 ± 0.30	0.70 ± 0.03^{d}	0.19 ± 0.01^{b}	1.03 ± 0.04^{e}	0.05 ± 0.00^{a}	nd	nd	0.62 ± 0.02^{c}	0.37 ± 0.38
Cytidine	3.13 ± 0.08^{b}	nd	3.09 ± 0.10^{b}	nd	nd	nd	10.65 ± 0.28^{d}	nd	5.25 ± 0.19^{c}	nd	1.44 ± 0.06^{a}	1.29 ± 0.06^{a}	2.07 ± 3.20	nd	nd	nd	nd	nd	nd	nd	–
Guanosine	9.55 ± 2.52^{c}	1.30 ± 0.05^{a}	9.60 ± 0.30^{c}	2.55 ± 0.10^{b}	nd	1.74 ± 0.07^{a}	nd	nd	nd	nd	13.13 ± 0.41d	nd	3.16 ± 4.74	nd	nd	nd	nd	nd	nd	nd	–
Guanosine-5′-monophosphate	0.61 ± 0.03^{b}	2.40 ± 0.09^{d}	2.02 ± 0.07^{c}	0.74 ± 0.04^{b}	0.57 ± 0.03^{ab}	nd	3.68 ± 0.12^{e}	3.61 ± 0.15^{e}	nd	nd	nd	0.40 ± 0.02^{a}	1.17 ± 1.40	nd	0.72 ± 0.04^{a}	1.32 ± 0.05^{d}	1.08 ± 0.05^{c}	0.84 ± 0.03^{b}	nd	nd	0.56 ± 0.52
Total	23.26 ± 0.70^{g}	9.53 ± 0.33^{c}	34.53 ± 0.99^{j}	11.39 ± 0.42^{d}	9.85 ± 0.37^{cd}	5.50 ± 0.24^{a}	32.22 ± 0.88^{i}	19.34 ± 0.68^{f}	15.00 ± 0.54^{e}	3.78 ± 0.15^{a}	28.99 ± 0.88^{h}	7.29 ± 0.27^{b}	16.72 ± 10.75	7.25 ± 0.27^{d}	5.38 ± 0.20^{b}	11.19 ± 0.41^{e}	2.17 ± 0.10^{a}	2.49 ± 0.10^{a}	6.13 ± 0.21^{c}	5.13 ± 0.21^{b}	5.68 ± 2.82

Sample no. is same as Table1; Means within a line with different superscripts differ significantly ($P < 0.05$); nd = not detected. Thymidine, 2′-deoxyuridine, 2′-deoxyinosine, 2′-deoxyadenosine-5′-monophosphate, adenosine-5′-monophosphate, inosine-5′-monophosphate and cytidine-5′-monophosphate were not detected in all the samples.

Table S8. Regression equation, correlation coefficients, linearity ranges and limit of detection (LOD) of the investigated fatty acids.

Analyte	Calibration curves [a]	r^2	Linear range (ng)	LOD (ng)
Octanoic acid	y = 23.425x + 0.0045	0.9997	0.6575-65.75	0.1315
Decanoic acid	y = 28.983x + 0.0061	0.9993	0.6575-65.75	0.1315
Dodecanoic acid	y = 33.007x - 0.0065	0.9998	0.6575-65.75	0.1315
Tridecanoic acid	y = 34.338x - 0.0222	0.9976	0.6575-65.75	0.1315
Palmitic acid	y = 69.039x - 0.0732	0.9965	1.315-131.5	0.3288
Linoleic acid	y = 23.079x - 0.0813	0.9961	3.288-65.75	0.6575
Linolenic acid	y = 26.349x - 0.0963	0.9968	3.288-65.75	0.6575
Oleinic acid	y = 13.522x - 0.0355	0.9976	3.288-65.75	0.6575
Stearic acid	y = 33.762x - 0.0682	0.9961	3.288-65.75	0.6575
Eicosenoic acid	y = 30.993x - 0.1525	0.9982	3.288-65.75	0.6575
Eicosanoic acid	y = 32.989x - 0.1023	0.9950	3.288-65.75	0.6575
Docosanoic acid	y = 30.772x - 0.1687	0.9987	3.288-65.75	0.6575

[a] y is the ratio of the peak area of reference substance to internal standard, and x is the value of the reference compound's amount (ng).

Table S9. Precision, repeatability, stability and recovery of the investigated fatty acids.

Analyte	Precision (RSD, %)		Repeatability (RSD, %, n=6)	Stability (RSD, %, n=6)	Recovery (%, n=3)	
	Intraday (n=6)	Interday (n=6)			Mean	RSD,%
Octanoic acid	1.12	2.98	2.98	2.24	104.0	3.10
Decanoic acid	1.28	3.22	3.18	3.01	95.3	3.91
Dodecanoic acid	1.98	3.19	3.79	2.68	101.5	2.82
Tridecanoic acid	2.04	2.50	4.33	2.77	95.4	3.41
Palmitic acid	1.93	3.28	2.69	1.67	94.2	2.90
Linoleic acid	2.47	3.67	4.95	3.14	93.3	5.38
Linolenic acid	2.55	2.91	5.65	2.93	94.2	5.71
Oleinic acid	2.58	4.32	4.77	3.05	93.9	4.92
Stearic acid	1.89	3.22	2.21	1.48	95.4	4.71
Eicosenoic acid	2.33	2.97	4.95	3.22	96.4	5.12
Eicosanoic acid	2.01	3.17	4.32	2.38	94.3	4.98
Docosanoic acid	1.99	2.94	3.67	2.67	93.4	4.71

Table S10. The profiles of fatty acids (mg/g) in the seeds of *Z. jujuba* var. *spinosa* and *Z. mauritiana* (mean ± SD).

Sample no.	Fatty acids												TSFA	TMUFA	TPUFA	TFA
	C 8:0	C 10:0	C 12:0	C 13:0	C 16:0	C 18:2	C 18:3	C 18:1	C 18:0	C 20:1	C 20:0	C22:0				
ZS01	nd	nd	nd	nd	3.55 ± 0.13^{a}	39.45 ± 1.10^{b}	5.08 ± 0.16^{b}	43.26 ± 1.41^{b}	1.74 ± 0.07^{a}	nd	nd	nd	5.28 ± 0.20^{a}	43.26 ± 1.41^{b}	44.52 ± 1.26^{b}	93.06 ± 2.86^{b}
ZS02	nd	nd	nd	nd	6.74 ± 0.22^{cd}	80.44 ± 2.93^{ef}	8.05 ± 0.34^{cd}	68.15 ± 2.80^{d}	3.41 ± 0.13d^{e}	1.22 ± 0.05^{c}	1.15 ± 0.06^{c}	0.61 ± 0.03^{c}	11.91 ± 0.44^{f}	69.37 ± 2.86^{d}	88.50 ± 3.27^{e}	169.75 ± 6.57^{f}
ZS03	nd	nd	nd	nd	6.11 ± 0.25^{c}	62.34 ± 1.96^{d}	8.40 ± 0.26^{de}	68.76 ± 2.65^{d}	2.90 ± 0.09^{c}	nd	nd	nd	9.02 ± 0.34^{bc}	68.76 ± 2.65^{d}	70.74 ± 2.22^{d}	148.52 ± 5.21^{de}
ZS04	nd	nd	nd	nd	7.50 ± 0.30^{ef}	77.83 ± 3.18^{e}	9.99 ± 0.30f^{g}	78.57 ± 2.33^{ef}	3.75 ± 0.15^{ef}	nd	nd	0.55 ± 0.03b	11.81 ± 0.48^{ef}	78.57 ± 2.33^{e}	87.82 ± 3.48^{e}	178.20 ± 6.29^{f}
ZS05	nd	nd	nd	nd	6.11 ± 0.15^{c}	73.10 ± 2.83^{e}	7.77 ± 0.40^{cd}	70.13 ± 3.52^{d}	3.69 ± 0.16^{ef}	1.15 ± 0.05^{c}	nd	nd	9.80 ± 0.31^{cd}	71.28 ± 3.57^{de}	80.86 ± 3.23^{e}	161.95 ± 7.10^{ef}
ZS06	nd	nd	nd	nd	4.80 ± 0.10^{b}	52.84 ± 1.70c	6.02 ± 0.30^{b}	50.16 ± 1.79^{bc}	2.45 ± 0.12^{b}	0.77 ± 0.03^{a}	0.74 ± 0.03^{a}	nd	7.99 ± 0.25^{b}	50.93 ± 1.82^{bc}	58.86 ± 2.00^{c}	117.78 ± 4.08^{c}
ZS07	nd	nd	nd	nd	7.24 ± 0.30^{de}	75.43 ± 3.02^{e}	9.09 ± 0.43^{ef}	75.93 ± 3.14^{de}	4.03 ± 0.13^{fg}	1.23 ± 0.06^{c}	1.15 ± 0.05^{c}	nd	12.42 ± 0.48^{f}	77.16 ± 3.20d^{e}	84.52 ± 3.44^{e}	174.07 ± 7.12^{f}
ZS08	0.22 ± 0.01a	0.18 ± 0.01a	0.19 ± 0.01a	0.25 ± 0.01b	10.00 ± 0.37^{h}	102.88 ± 2.79^{h}	10.52 ± 0.42^{g}	93.96 ± 3.45^{g}	5.47 ± 0.23^{i}	nd	nd	nd	16.31 ± 0.72^{h}	93.96 ± 3.45^{f}	113.41 ± 3.21^{g}	223.68 ± 7.37^{h}
ZS09	nd	nd	nd	nd	3.48 ± 0.10^{a}	29.85 ± 1.09^{a}	3.76 ± 0.09^{a}	32.31 ± 1.32^{a}	1.66 ± 0.06^{a}	nd	nd	nd	5.13 ± 0.16^{a}	32.31 ± 1.32^{a}	33.62 ± 1.18^{a}	71.06 ± 2.66^{a}
ZS10	nd	nd	nd	nd	6.19 ± 0.23^{c}	61.09 ± 2.08^{d}	7.11 ± 0.24^{c}	57.75 ± 2.42^{c}	3.09 ± 0.11^{cd}	0.92 ± 0.04b	0.88 ± 0.05^{b}	0.34 ± 0.01^{a}	10.49 ± 0.40d^{e}	58.66 ± 2.46^{c}	68.20 ± 2.33^{d}	137.35 ± 5.18^{d}
ZS11	nd	nd	nd	nd	8.52 ± 0.36^{g}	87.66 ± 3.39^{fg}	11.59 ± 0.36^{h}	92.48 ± 3.59^{g}	4.47 ± 0.19h	1.62 ± 0.07d	1.56 ± 0.08^{d}	0.63 ± 0.03^{c}	15.18 ± 0.65^{gh}	94.10 ± 3.65^{f}	99.25 ± 3.75^{f}	208.54 ± 8.06^{gh}
ZS12	nd	0.04	nd	0.07 ± 0.01a	8.17 ± 0.30^{fg}	88.38 ± 3.83^{g}	11.66 ± 0.50^{h}	86.02 ± 4.05^{fg}	4.31 ± 0.22^{gh}	1.67 ± 0.05^{d}	1.62 ± 0.06^{d}	0.81 ± 0.04^{d}	15.02 ± 0.62^{g}	87.69 ± 4.10^{f}	100.05 ± 4.33^{f}	202.78 ± 9.05^{g}
Mean	0.02 ± 0.06	0.02 ± 0.07	0.02 ± 0.05	0.03 ± 0.07	6.53 ± 1.90	69.27 ± 20.63	8.25 ± 2.44	68.12 ± 18.86	3.41 ± 1.10	0.72 ± 0.66	0.59 ± 0.64	0.25 ± 0.31	10.87 ± 3.57	68.84 ± 19.14	77.53 ± 22.91	157.24 ± 45.27
ZM01	nd	0.05 ± 0.01	nd	0.15 ± 0.00a	11.72 ± 0.51^{a}	37.54 ± 1.37^{c}	8.64 ± 0.35^{b}	67.18 ± 2.80^{b}	8.49 ± 0.41^{b}	1.04 ± 0.04^{a}	0.98 ± 0.05^{a}	1.82 ± 0.08^{b}	23.22 ± 1.05^{b}	68.21 ± 2.84^{b}	46.18 ± 1.72^{c}	137.61 ± 5.61^{b}
ZM02	nd	nd	nd	nd	18.35 ± 0.58^{b}	41.08 ± 1.23^{d}	8.87 ± 0.43^{bc}	78.58 ± 3.95^{cd}	11.88 ± 0.62^{d}	nd	nd	nd	30.23 ± 1.20^{c}	78.58 ± 3.95^{cd}	49.95 ± 1.66^{c}	158.76 ± 6.81^{c}
ZM03	nd	nd	nd	nd	10.98 ± 0.44^{a}	32.52 ± 1.27^{ab}	7.06 ± 0.25^{a}	63.75 ± 3.29^{b}	6.56 ± 0.20^{a}	nd	nd	nd	17.54 ± 0.64^{a}	63.75 ± 3.29^{b}	39.58 ± 1.52^{ab}	120.87 ± 5.46^{a}
ZM04	nd	nd	nd	nd	20.48 ± 0.57^{c}	40.47 ± 0.99^{cd}	9.66 ± 0.31^{c}	83.05 ± 4.13^{de}	12.97 ± 0.39^{d}	nd	nd	nd	33.45 ± 0.96^{d}	83.05 ± 4.13^{de}	50.13 ± 1.30^{c}	166.63 ± 6.38^{cd}
ZM05	0.34 ± 0.01^{a}	0.28 ± 0.01^{a}	0.36 ± 0.01^{a}	nd	18.23 ± 0.79^{b}	33.69 ± 1.38^{b}	7.68 ± 0.28^{a}	70.38 ± 2.56^{bc}	10.14 ± 0.38^{c}	nd	nd	1.00 ± 0.04^{a}	30.35 ± 1.42^{c}	70.38 ± 2.56^{bc}	41.38 ± 1.65^{b}	142.11 ± 5.64^{b}
ZM06	nd	nd	nd	nd	11.60 ± 0.40a	29.78 ± 1.14^{a}	6.79 ± 0.19^{a}	48.96 ± 1.85^{a}	7.62 ± 0.31^{ab}	nd	nd	0.91 ± 0.05^{a}	20.13 ± 0.76^{a}	48.96 ± 1.85^{a}	36.56 ± 1.33^{a}	105.66 ± 3.94^{a}
ZM07	nd	nd	nd	0.28 ± 0.00b	20.43 ± 0.60c	46.58 ± 1.00^{e}	10.95 ± 0.47^{d}	88.26 ± 3.72^{e}	13.04 ± 0.50^{d}	nd	nd	1.79 ± 0.08^{b}	35.54 ± 1.19^{d}	88.26 ± 3.72^{e}	57.53 ± 1.47^{d}	181.33 ± 6.38^{d}
Mean	0.05 ± 0.12	0.06 ± 0.16	0.05 ± 0.13	0.06 ± 0.11	15.97 ± 4.15	37.38 ± 5.61	8.52 ± 1.44	71.45 ± 12.86	10.10 ± 2.51	0.15 ± 0.37	0.14 ± 0.35	0.79 ± 0.77	27.22 ± 6.63	71.60 ± 12.81	45.90 ± 7.01	144.72 ± 25.47

Sample no. is same as Table1; Means within a line with different superscripts differ significantly ($P < 0.05$); nd = not detected. Octanoic acid (C 8:0), decanoic acid (C 10:0), dodecanoic acid (C 12:0), tridecanoic acid (C 13:0), palmitic acid (C 16:0), linoleic acid (C 18:2), linolenic acid (C 18:3), oleic acid (C 18:1), stearic acid (C 18:0), eicosenoic acid (C 20:1), eicosanoic acid (C 20:0), docosanoic acid (C 22:0); TSFA, TMUFA, TPUFA and TTFA refer to the total content of SFA, MUFA, PUFA and fatty acids, respectively

Table S11. The data detected in the samples and used for multivariate PCA and OPLS-DA analyses.

No.	Analytes	Seeds of *Ziziphus jujuba* var. *spinosa*												Seeds of *Ziziphus mauritiana*						
		ZS01	ZS02	ZS03	ZS04	ZS05	ZS06	ZS07	ZS08	ZS09	ZS10	ZS11	ZS12	ZM01	ZM02	ZM03	ZM04	ZM05	ZM06	ZM07
Flavonoids and triterpenoids (mg/100 g)																				
1	Spinosin	84.02	77.88	61.20	21.98	89.36	31.29	25.94	90.51	69.84	79.25	113.64	132.38	107.48	128.22	78.84	94.04	67.64	124.88	62.97
2	6'''-feruloylspinosin	22.22	42.20	21.55	15.32	26.07	11.66	26.52	42.56	39.55	35.78	45.75	40.48	34.47	42.71	29.26	18.59	20.96	48.91	23.23
3	Jujuboside A	79.66	70.84	37.12	74.78	76.55	89.03	55.05	78.47	59.15	90.34	114.08	125.41	nd	nd	nd	nd	nd	nd	nd
4	Jujuboside B	29.04	19.63	14.64	11.89	10.05	19.20	13.71	20.59	18.17	13.69	16.43	23.80	nd	nd	nd	nd	nd	nd	nd
5	Betulinic acid	38.56	63.76	31.54	36.24	37.37	51.91	72.41	50.27	55.30	90.51	92.57	89.41	31.47	49.52	27.35	31.09	48.50	92.60	40.01
Nucleosides and nucleobases (mg/100 g)																				
6	Thymine	0.17	nd	0.14	nd	nd	nd	nd	nd	nd	nd	0.22	nd	nd	nd	nd	nd	nd	nd	nd
7	2′-deoxyadenosine	0.26	0.05	0.25	0.07	0.03	0.01	0.06	0.05	0.13	0.01	0.33	0.02	0.02	0.01	0.09	nd	nd	0.07	0.12
8	Adenine	2.95	1.42	5.07	2.17	2.28	1.33	4.48	2.80	2.82	0.63	3.68	1.27	0.80	0.54	0.69	0.27	0.50	2.56	0.90
9	Uridine	3.22	1.41	7.25	2.93	3.92	0.65	3.88	5.24	2.60	1.77	6.12	1.69	2.60	1.77	2.28	nd	0.69	2.75	1.27
10	Hypoxanthine	0.34	0.11	0.58	0.18	0.23	0.15	0.28	2.75	0.45	0.11	0.41	0.16	0.79	0.36	4.25	nd	0.07	0.70	0.78
11	Adenosine	3.02	2.77	6.49	2.74	2.76	1.59	9.20	3.82	3.44	1.07	3.63	2.38	2.32	1.78	1.53	0.78	0.39	0.04	1.32
12	Inosine	nd	0.05	nd	nd	0.06	0.02	nd	1.05	0.31	0.20	0.02	0.09	0.70	0.19	1.03	0.05	nd	nd	0.62
13	Cytosine	0.01	0.01	0.03	0.01	nd	0.01	nd	0.01	nd	nd	0.02	nd	0.02	0.02	nd	nd	nd	0.01	0.12
14	Cytidine	3.13	nd	3.09	nd	nd	nd	10.65	nd	5.25	nd	1.44	1.29	nd	nd	nd	nd	nd	nd	nd
15	Guanosine	9.55	1.30	9.60	2.55	nd	1.74	nd	nd	nd	nd	13.13	nd	nd	nd	nd	nd	nd	nd	nd
16	Guanosine-5′-monophosphate	0.61	2.40	2.02	0.74	0.57	nd	3.68	3.61	nd	nd	nd	0.40	nd	0.72	1.32	1.08	0.84	nd	nd
Amino acids (mg/100 g)																				
17	Leucine	10.60	5.70	28.50	8.70	7.90	5.40	11.70	14.90	36.80	2.60	21.20	4.20	3.60	1.50	4.50	0.30	0.40	0.80	1.10
18	Phenylalanine	7.60	4.50	19.10	6.60	7.80	5.10	11.10	12.30	27.00	2.40	13.90	4.20	3.60	1.80	4.20	0.40	0.50	1.20	1.70
19	Isoleucine	6.90	4.40	19.50	6.90	5.80	3.90	7.90	11.10	18.30	1.80	12.20	3.70	2.20	1.20	1.70	0.60	0.50	1.20	1.30
20	Tryptophan	3.60	2.20	7.20	2.70	3.60	1.80	5.90	6.40	9.70	1.30	5.20	2.00	2.10	1.10	1.30	0.30	0.70	1.80	1.20
21	γ-aminobutyric acid	6.80	6.90	40.40	20.70	4.10	6.60	9.40	26.10	11.20	1.10	23.80	5.30	1.30	0.60	0.60	nd	0.10	0.20	1.20
22	Methionine	3.50	2.50	6.70	3.60	2.00	1.90	2.90	2.60	6.90	0.30	4.90	0.70	1.00	nd	0.70	nd	nd	nd	0.50
23	Proline	14.40	8.90	33.20	20.80	17.40	7.20	15.40	13.60	30.50	2.40	25.90	4.40	2.90	1.90	1.80	0.60	0.80	1.60	nd
24	Valine	10.40	7.00	28.30	10.50	8.20	6.20	10.80	15.50	31.00	2.60	18.10	5.20	4.40	1.90	3.10	0.40	0.30	1.70	1.50
25	Tyrosine	19.20	20.20	44.40	30.30	19.80	18.30	38.40	46.50	62.40	13.40	34.90	16.20	6.50	4.80	3.50	nd	1.70	nd	nd
26	Alanine	19.90	16.50	46.80	22.10	9.70	17.70	16.10	23.00	34.30	5.50	26.10	5.10	3.70	2.10	7.40	1.80	3.00	3.00	nd

27	Hydroxyproline	nd	nd	nd	0.60	nd	nd	nd	nd	2.80	nd	nd	nd	0.70	nd	nd	nd	nd	nd	nd
28	Threonine	13.50	11.40	36.50	8.00	3.60	8.50	7.50	8.20	26.50	0.70	9.60	1.80	2.70	1.30	2.80	nd	nd	nd	nd
29	Glutamic Acid	36.30	10.20	59.30	16.70	15.90	11.70	18.90	53.20	49.20	7.60	34.20	5.30	10.90	10.40	9.10	2.70	2.70	11.20	1.90
30	Lysine	16.20	11.70	29.00	13.70	11.70	21.20	7.40	30.40	115.80	9.30	16.20	4.90	2.50	6.10	4.80	nd	nd	nd	2.80
31	Glutamine	15.50	8.20	31.50	9.80	8.00	21.50	7.30	16.00	42.50	5.60	12.70	4.40	3.70	3.00	9.00	nd	nd	1.10	4.70
32	Serine	14.80	17.10	55.50	20.40	9.40	12.40	90.90	134.10	43.90	5.70	39.50	15.00	13.20	15.80	nd	nd	nd	nd	68.30
33	Asparagine	54.40	28.50	99.30	45.40	19.50	73.10	72.30	92.40	65.30	24.20	57.40	12.00	7.90	nd	13.60	nd	nd	nd	nd
34	Citrulline	1.60	nd	nd	1.20	nd	nd	nd	nd	nd	nd	2.50	1.10	nd	nd	nd	nd	nd	nd	nd
35	Arginine	30.90	23.20	131.60	73.80	54.40	52.20	121.40	166.70	283.40	38.70	153.60	33.90	47.10	16.70	47.50	7.90	10.40	50.10	45.30
36	Histidine	9.30	7.40	29.90	15.10	10.40	10.60	14.40	23.30	25.70	4.50	16.60	5.80	9.00	4.60	6.20	1.80	0.90	2.40	6.40
37	Ornithine	nd	nd	2.20	nd	nd	1.70	nd	nd	nd	nd	0.80	4.90	nd	nd	nd	nd	nd	nd	nd
Fatty acids (mg/g)																				
38	Octanoic acid	nd	nd	nd	nd	nd	nd	nd	0.22	nd	nd	nd	nd	nd	nd	nd	nd	0.34	nd	nd
39	Decanoic acid	nd	nd	nd	nd	nd	nd	nd	0.18	nd	nd	nd	0.04	0.05	nd	nd	nd	0.28	nd	nd
40	Dodecanoic acid	nd	nd	nd	nd	nd	nd	nd	0.19	nd	nd	nd	nd	nd	nd	nd	nd	0.36	nd	nd
41	Tridecanoic acid	nd	nd	nd	nd	nd	nd	nd	0.25	nd	nd	nd	0.07	0.15	nd	nd	nd	nd	nd	0.28
42	Palmitic acid	3.55	6.74	6.11	7.50	6.11	4.80	7.24	1nd	3.48	6.19	8.52	8.17	11.72	18.35	10.98	20.48	18.23	11.60	20.43
43	Linoleic acid	39.45	80.44	62.34	77.83	73.10	52.84	75.43	102.88	29.85	61.09	87.66	88.38	37.54	41.08	32.52	40.47	33.69	29.78	46.58
44	Linolenic acid	5.08	8.05	8.40	9.99	7.77	6.02	9.09	10.52	3.76	7.11	11.59	11.66	8.64	8.87	7.06	9.66	7.68	6.79	10.95
45	Oleinic acid	43.26	68.15	68.76	78.57	70.13	50.16	75.93	93.96	32.31	57.75	92.48	86.02	67.18	78.58	63.75	83.05	70.38	48.96	88.26
46	Stearic acid	1.74	3.41	2.90	3.75	3.69	2.45	4.03	5.47	1.66	3.09	4.47	4.31	8.49	11.88	6.56	12.97	10.14	7.62	13.04
47	Eicosenoic acid	nd	1.22	nd	nd	1.15	0.77	1.23	nd	nd	0.92	1.62	1.67	1.04	nd	nd	nd	nd	nd	nd
48	Eicosanoic acid	nd	1.15	nd	nd	nd	0.74	1.15	nd	nd	0.88	1.56	1.62	0.98	nd	nd	nd	nd	nd	nd
49	Docosanoic acid	nd	0.61	nd	0.55	nd	nd	nd	nd	nd	0.34	0.63	0.81	1.82	nd	nd	nd	1.00	0.91	1.79

nd = not detected.
